# Data survey on the effect of product features on competitive advantage of selected firms in Nigeria

**DOI:** 10.1016/j.dib.2018.03.134

**Published:** 2018-04-04

**Authors:** Maxwell Olokundun, Oladele Iyiola, Stephen ibidunni, Hezekiah Falola, Odunayo Salau, Augusta Amaihian, Fred Peter, Taiye Borishade

**Affiliations:** Covenant University, Nigeria

**Keywords:** Product features, Competitive advantage, Small business owners, Nigeria

## Abstract

The main objective of this study was to present a data article that investigates the effect product features on firm's competitive advantage. Few studies have examined how the features of a product could help in driving the competitive advantage of a firm. Descriptive research method was used. Statistical Package for Social Sciences (SPSS 22) was engaged for analysis of one hundred and fifty (150) valid questionnaire which were completed by small business owners registered under small and medium scale enterprises development of Nigeria (SMEDAN). Stratified and simple random sampling techniques were employed; reliability and validity procedures were also confirmed. The field data set is made publicly available to enable critical or extended analysis.

**Specification Table**

**Table:** 

**Subject area**	Business, Management
**More Specific Subject Area:**	Business Administration
**Type of Data**	Table
**How Data was Acquired**	Researcher-made questionnaire analysis
**Data format**	Raw, analyzed, Inferential statistical data
**Experimental Factors**	Sample consisted of selected small business owners in Lagos, Nigeria. The researcher-made questionnaire which contained data on product features and competitive advantage were completed..
**Experimental features**	Competitive advantage is an imperative element of organizational performance in modern-day organizations
**Data source location**	South west Nigeria
**Data Accessibility**	Data is included in this article

**Value of data**

• These data present information on product features as it relates to a firm's competitive advantage. This is important considering that developing a competitive advantage means that a firm's brand name becomes recognized in the marketplace as being the best.
• The results showed that the potency of a firm's brand name stemming from the uniqueness of product features contributes to a firm's competitive advantage.
• These results can motivate product differentiation activities within a firm in order to enhance the development of unique products and firm's competitive advantage.

## 1 Data

The data comprised raw inferential statistical data on the effect of product features on competitive advantage of small businesses in Lagos, Nigeria. Specifically, regression analysis was used to test the effect of the independent variable on the dependent variable. Table 1 shows the model summary of the analysis based on the hypothesis tested. It shows how much variance in the dependent variable (competitive advantage) is explained by the independent variable (product features).

H_**0**_ _**:**_ There is no significant relationship between product features and competitive advantage.

**Table 1:** Model summary.

Model	R	R Square	Adjusted R Square	Standard Error of the Estimate
1	.671(a)	.450	.446	.43659

a Predictors: (Constant), product features

Table 2 below shows the assessment of the statistical significance of the result. The Analysis of variance table tests the null hypothesis to determine if it is statistically significant. From the results, the model appears to have a good fit, indicated by positive *F* value of 110.413. Also, the table shows a statistically significant relationship between product feature and competitive advantage (*p* < 0.01).

**Table 2:** Analysis of variance.

Model		Sum of squares	Df	Mean square	F	Sig.
1	Regression	21.045	1	21.045	110.413	.000(a)
	Residual	25.732	135	.191		
	Total	46.777	136			

a Predictors: (Constant), product features

b Dependent Variable: competitive advantage

Table 3 below also shows the contribution of the independent variable to the prediction of the dependent variable. In the table, the beta co-efficient is .671, which relates to product features. It makes a strong contribution to explaining the dependent variable.

**Table 3:** Coefficients.

Model		Unstandardized coefficients		Standardized coefficients	T	Significance
		B	Standard error	Beta	B	Standard error
1	(Constant)	.952	.320		2.973	.003
	Product features	.780	.074	.671	10.508	.000

Dependent Variable: competitive advantage

## 2 Experimental design, materials and methods

The data presented was based on a quantitative study. Descriptive research design was adopted to examine the effect of product features on firm's competitive advantage. Survey method was considered suitable as data collection method. Small businesses were selected from Lagos State, South west Nigeria. The study population included small business owners in Lagos State registered under Small and Medium Scale Enterprises Development of Nigeria (SMEDAN), which has a population of 4535 businesses. 150 business owners were selected to participate in this study. Data were collected from small business owners across the Mainland and Island in Lagos State Nigeria, using a researcher- made questionnaire [2], [4], [5]. The collected data were coded and entered into SPSS version 22. Data analysis was done; using Statistical Package for Social Sciences-22 [1], [3]. Data was analyzed using inferential statistical tests which involved regression analysis.

Even though Statistical Package for Social Sciences may be restricted as regards complex modeling in statistics however, it makes data analysis easier, faster and accurate. More significantly, Statistical Package for Social Sciences is designed to certify that the output is set aside separately from the data itself especially because it stores all results in a separate file different from the data. This facilitates further analysis if required. The researchers ascertained that the respondents were adequately informed about the purpose of this research and they were kept abreast with the participation process and administration. Respondents were given the opportunity to stay unidentified and their responses were treated confidentially. Permission was obtained from the appropriate authorities in the organisations prior to the distribution of the copies of questionnaire.

## References

[1] Olokundun M.A., Ibidunni A.S., Peter F., Amaihian A.B., Ogbari M. (2017). Entrepreneurship educator's competence on university students' commitment to learning and business plan writing. Acad. Strat. Manag. J..

[2] Olusanya S.O., Adegbola E.A. (2014). Impact of total quality management practice on small and medium scale enterprises in Nigeria. (A Case Study of Small Business Owners in Lagos). J. Bus. Manag..

[3] Osibanjo O.A., Salau O.P., Falola H.O., Oyewunmi A.E. (2016). Workplace stress: implications for organizational performance in a Nigerian Public University Business. Theory Pract..

[4] Oua C.S., Liua F.C., Hunga Y.C., C, D. Y (2012). The effects of total quality management on business performance: evidence from Taiwan information-related industries. J. Decis. Sci. Manag. Inf. Syst..

[5] Oua C.S., Liua F.C., Hunga Y.C., David Y.C. (2012). The effects of total quality management on business performance: evidence from Taiwan information-related industries. J. Decis. Sci. Manag. Inf. Syst..

