# Supplementary material for: Data survey on the effect of product features on competitive advantage of selected firms in Nigeria
Source: Data Brief. 2018 Apr 4;18:1005–8. doi: 10.1016/j.dib.2018.03.134 (PMC5996723; doi:10.1016/j.dib.2018.03.134)
Supplement: Supplementary file 2 — Supplementary material [file mmc2.docx]

**IMPACT OF PRODUCT QUALITY MANAGEMENT ON THE GROWTH OF SMALL AND MEDIUM SCALE ENTERPRISES IN NIGERIA: A STUDY OF SELECED SMALL BUSINESS IN LAGOS STATE.**

***ABSTRACT***

*It is evident that product quality management leads to the growth of small and medium scale enterprises, this has been evident in the market share , profitability level and sustainability of most small business that has adopted this concept. This study was adopted based on questionnaires administered to selected small businesses, in Lagos,150 questionnaires were distributed to the respondents Regression analysis and analysis of variance were used to test these hypotheses through the statistical package for social science .Results of data analysis showed that there exist positive relationship between product quality management and small businesses. The result further revealed that product futures, continuous improvement, perceived quality and product durability are important antecedent factors that determine the success or implementation of quality management. From this study it was concluded that business who adopt product quality management techniques will have competitive advantage over its rivals and enjoy growth and overall sustainability. The study recommends quality management is the responsibility of everyone in the organization and not just the manufacturer, the employees should be properly enlightened about this concept. In addition, the customers suppliers and other participants in the internal and external business environment should be considered as important factors in the practice of quality management, so the government should provide necessary support needed by SME’s to achieve quality goals.*

**CHAPTER ONE**

**INTRODUCTION**

- 1. **BACKGROUND OF THE STUDY**

Over time the adoption of quality management in both small and large firms has lead to increased success in the areas of profitability increased market share and so on (Collins, 2012). The advert of globalization has resulted in increased competition amongst firms, as such managers of today are increasingly seeking different ways and approaches to achieve, improve, and sustain organizational performance and competitive advantage in today’s competitive business environment, (Ivaničková, 2014), this suggest that every firm is expected to the best approach towards ensuring long lasting survival in the market, the influence product quality management has on small and medium scale enterprise is widely recognized and seen as a growth and survival strategy in the dynamic environment (Egbuogu, 2003). SME, being the back bone of every economy cannot be over emphasized because the performance of small business to a large extent determines the level of growth of an economy and has a positive impact on the Gross Domestic Product (GDP) and on the creation of jobs the economy (Jones,2013)

A number of studies have concluded that the practice and implementation of good management is a key ingredient to a firm’s success (Ghosh, Teo, and Low, 1993; Yusuf, 1995). To drive the manufacturing and other sectors into accelerated growth and global competitiveness, quality management allows organization to keep up and meet the current and emerging needs of consumers for quality and keep up with the latest technology. (Bennett and Vaidya, 2001). According to the American Society for Quality, “quality” can be defined in the following ways:

- Based on customer’s perceptions of a product/service’s design and how sound the design matches the original specifications.
- The ability of a product/service to suit stated or implied needs.

This are Achieved by conforming to established requirements within an organization.

According to The Bolton Committee (1971) a small business is an independent firm, which has relatively small share of its market place, and managed by the owners themselves, or part owners personally, and not by the medium of formalized management structure. There is hardly any unique, universally accepted definition of SME because the classification of business into small and large scale is a subjective judgment (Ekpeyong and Nyong, 1992). Egbuogu (2003), noted that definitions of SME’s vary both between countries and continents. The major criteria used in the definition Small businesses according to carpenter (2003) could include various combinations of the following: Number of employees, financial strength, Sales value, Relative size, Initial capital outlay and Types of industry. The product quality management concept was developed by a number of American management consultants, including Feigenbaum (1991). It is seen as a continuous process because customer taste and preference changes and in order to enjoy continuous patronage; the quality of your product must be constantly improved.

- 1. **STATEMENT OF THE PROBLEM**.

Small business are known for being good with creativity and innovativeness, because they are still young, even with this they still lack behind in the adoption of quality (Wanjuau, Kahiri,and Gakure n.d), (McMahon, 2001). The relevance of quality management in small business is seen as very important because the success of a business can be measured to the degree in which a firm is able to produce higher quality goods or services than competitors (Lobo, and Jones 2013). Quality management practice has been proposed to improve business performance the effect has been reflected on reduced cost improved level of profit and has guaranteed a competitive edge over competitors. (Oua, Liua, Hunga, and David, 2012). Despite the cited benefits, a large percentage of SME are faced with challenges when it comes to the practice of product management. One problem is that the implementation of the initiatives in small businesses is said to be inadequate (Ramsey 1998; Kuratko, Goodale and Hornsby, 2001). Small businesses have been very slow to implement the formal product management, (Lobo, and Jones (2013). This can be as a result of inadequate knowledge about implementation and resources.

Another problem faced is less focus on product quality by the management, rather their day to day activities centers on how to increase sales, and customer base, forgetting that the first focus should be on the product itself (Yusof and Aspinwall, 1999; McTeer and Dale, 1994). For business to succeed and achieve its best result the first strategy to be taken is quality on whatever good or services produced, this will have a contributing factor on the business growth. If this problem is addressed the first problem mentioned will be curtailed. For some business a problem faced is unawareness of the part of the product to improve on in terms of packing and branding e.t.c. if the small business is not able to identify the part to improve on, the needs of the consumers will not be met (Austrian Competition and Consumer Commission, 2012). Lack of commitment on continuous improvement of the product is seen as another problem of product quality management in small businesses, (Tjosvold and Tjosvold, 1995). Once a product has been improved upon it is vital to take improvement as a continuous process but most SME’s are lagging behind in this area, this can be said to be as a result of lack of understanding of the necessity of product quality improvement and the influence it has on the business (Teece, 2010). Regardless of the level adoption of product quality management in ensuring that a business remains outstanding in its performance, some organizations still believe that customers will still purchase a product in its current state, this should not be so rather there should be need for improvement.

Finally, recent contributors to literature have made it clear that there has been a fall overtime in the growth of small businesses in Nigeria, due to inadequate resources poor financing and lack of implementation of specific strategies to achieve market growth (Internatioal Monetary Fund, 2010). Research has shown that the sales revenue of small business has been reducing by 5%-15% as a result of lack of attention to quality (McMahon, 2001). This shows that quality management is a vital tool in contributing to the sales thereby leading to growth of SME. This study therefore is aimed at addressing the gap in research and pinpointing the relationship between product quality management and growth in SME’s.

- 1. **OBJECTIVES OF STUDY**

The primary objective of the study is to assess the impact of product quality management on the growth of small business; this examines how product quality affects the growth of small businesses in Nigeria, using Lagos state as a study with an attempt to achieve the following objectives:

1. To determine whether the features of a product can lead to competitive advantage in small businesses.
2. To examine the relationship between continuous improvement product and small business sustainability.
3. To evaluate the impact of perceived quality on small business profitability.
4. To ascertain how product durability can lead to increased market share.
   1. **RESEARCH QUESTIONS**

Research questions will be used to derive valuable information and details on product quality and small businesses growth.

This research seeks to provide answers to the following questions.

1. Can the features of product lead to competitive advantage in small businesses?
2. Is there a link between continuous improvement product and sustainability in small business?
3. How can the perceived quality of a product lead to profitability?
4. To what extent can product durability lead to increased market share?
   1. **RESEARCH HYPHOTHESES**

Hypothesis can simply be seen as a tentative statements of real facts that are subject to various empirical validation, experimentation and testing used as a basis for further study and investigation. The following hypothesis listed below is to be tested.

**HYPOTHESIS 1**

**H_0:_** There is no significant relationship between product features and competitive advantage.

**H_1:_** There is a significant relationship between product features and competitive advantage.

**HYPOTHESIS 2**

**H_0:_** There is no link between continuous improvement and sustainability in small business.

**H_1:_** There is a link between continuous improvement and sustainability in small business.

**HYPOTHESIS 3**

**H_0:_** There is no significant relationship between perceived quality and profitability.

**H_1:_** There is a significant relationship between perceived quality and profitability.

**HYPOTHESIS 4**

**H_0:_** Product durability is not a measure of market share.

**H_1:_** Product durability is a measure of market share.

- 1. **SIGNIFICANCE OF THE STUDY**

This study is immensely significant in diverse ways to small businesses. The findings and results that will be reported in this study will provide a more comprehensive outlook on the impact product quality management has on small and medium enterprises. Authors and researchers who has carried out these studies, especially those on small firms, have drawn conflicting conclusions, some claim that product quality management has influence more on larger firms than small firms accompanied with increased market share and profitability as a result of its uniqueness in reliability and quality, while others conclude that product quality management has influence on both small and large firms generally. The results of this study is most likely useful to other researchers, consultants, academicians, stakeholders, policy makers, firms and managers generally. This research on their quest on the impact of product quality will help to increase product features and adopt more strategies to increase competitive advantage and increase the level of repeat purchase which will lead to increased profitability and will invariably as a small business play a positive role in the growth of the economy at large.

- 1. **OPERATIONALIZATION OF RESEARCH VARIABLES**

Operationalisation is the process of transforming a person’s theoretical project into an equation or mathematical concept. In operationalization of research variables, two variables are identified as “the dependent variable” and “the independent variable”.

In this study the dependent variable here is ‘’product quality management’’, and the independent variable is ‘’growth of small businesses’’.

That is, Y is a function of X

Mathematically, Y= *f* (x)

Dependent variable (y) and independent variable (x)

Here Y=$f$(X)

Y= product quality

X= growth of small business

: product quality=$f($growth of small business)

Variables of product quality include; PQ= (X_1_,X_2,_ X_3,_ X_4….._x_n_ )

Where,

X_1_= Product features

X_2_= continuous improvement of Products

X_3_= Perceived quality

X_4_ = Product durability

Variables of growth of small business include; GSB= (Y_1,_ Y_2,_ Y_3,_ Y_4….._Y_n_ )

Where ,

Y_1_ = Competitive advantage

Y_2_ = sustainability

Y_3_ = Profitability

Y_4_ = increased market share

- 1. **METHODOLOGY**

Methodology is the use of different methods and procedures to create scientifically based knowledge. It is constrained to objectives, procedures, and techniques that are relevant within the discipline. The research methodology here refers to the methods that entails the research instrument, the population of the study, the sample and the sampling techniques, the instrument for collection of data, the level of validity and the reliability, and then the method of presentation.

Data for this study will be obtained from both primary and secondary sources. The primary data is collected specifically for a particular purpose or particular research. Primary data is gotten through questionnaires, interviews and observation. The primary data in this research work would be gotten from questionnaires. Secondary data is data that already exists and is not collected for the specific purpose of the research project. Secondary data used in this research work would be gotten from journal articles, textbooks, Internet and unpublished project. In this study regression analysis was used to test the hypothesis.

- 1. **SCOPE OF THE STUDY**

The scope of the study is based on the impact of product quality management on the growth of small business in Nigeria with focus on Lagos state which is regarded as the business centre of Nigeria with a large population of small business.

- 1. **LIMITATIONS OF STUDY**

This study is limited by a number of variables. The exploratory nature of this research in addition to the usual problems that is always encountered in survey research techniques should be considered as some of the major limitations to the present study. Limited fund is also a challenge faced The cost involved in printing the project work, printing questionnaires, can be a major limitation to this study. Inability to access necessary data and information from respondent, and unavailability of important information/resources for the project work, Limited time to carry out research work due to the intensity of the work and credit load undertaken by the final year student, little time is left for the execution of the research project.

- 1. **OUTLINE OF CHAPTERS**

The research study is segmented into five different chapters. The first chapter is a general introduction to the study that comprises of the background, statement of the problem, objective of the study, research hypothesis, significance of the study, operationalization of variables, scope of study, limitation of study, outline of chapters and definition of terms. The second chapter reviews several literatures on conceptual framework empirical frame work and theoretical frame work. Chapter three focuses on research methods. Chapter four centers on the data presentation, analysis and interpretation and five focuses on the summary findings, conclusion and recommendation for future research study.

- 1. **DEFINITION OF TERMS**

1. Market share: This is the total percentage of an industry or market's total sales that is earned by a particular company over a specific time period. Market share is calculated by taking the company's sales over the period and dividing it by the total sales of the industry over the same period. This is used to give a general idea of the size of a company to its market and its competitors (investopedia, 2015).
2. Product durability: this can be defined as the amount of use a consumer gets from a particular product before it deteriorates David (1987).
3. Business growth: this is defined as the process of improving some measure of an enterprise success. Business growth can either be achieved by boosting the revenue of a business with greater product sale, or by increasing the profitability of the operation by minimizing cost (business dictionary, 2014).
4. Competitive advantage: it is an advantage a firm has over its competitors by producing more effective goods and services which allows it to generate greater revenue, or retain more customers than its competition in the same market (Boulter, 2013).
5. Consumers: Wikipedia defined a consumer as an individual who buys or goods or services for their own use.
6. Profitability**:** It is described as an income from an investment or business transaction. This could also be described as excess of income over expenditure especially in business (Feldblum, 1997).
7. Performance: the process of carrying out a task or function effectively, in a way that it is measurable in terms of output, market share or growth of an organization (Stepsis, 1998).
8. Products: A product can also be viewed as a totality of goods and service that a person or company makes available (Dictionary.com, 2015) pointed out that a product consists of both goods (tangible) and services (intangible), (Agwu, 2014).
9. Product Quality: describes how good and high the standard of a given product is, and this is determined by the standard of the raw materials that are used in its production process (Aw, Xie and Haemmerle, 2008) .

**CHAPTER TWO**

**LITERATURE REVIEW**

**2.0 Introduction**

This chapter looks thoroughly reviewed, the impact of product quality management on small businesses viewed by different authors who also have in the subject matter. It also examines the guiding theories and limiting factors involved in the interested area of study. A major and general assumption of literature review is that an in-depth knowledge of the background of study is highly important in other to make advancement in the study of interested area.

In this chapter of literature review, visited areas would be the basic characteristics of quality management and small businesses the history and concept of the subject matter , This research study carries out a comprehensive review as regards different dimensions of product quality management and the impact it would have on a small businesses. The product quality management variables are closely reviewed to demonstrate how managers can practice the concept well to the advantage of the business.

This literature would be analyzed under three dimensions which are: Conceptual Framework, Theoretical Framework, and Empirical framework.

**2.1 Conceptual Framework**

**2.1.0 PRODUCT QUALITY AS A TOTALITY OF GOODS AND SERVICES**

The only way a business is said to serve people in the market is through the manufacturing and availability of a product (Burnett, 2010). Many manufacturers and retailers tend to think only in terms of tangible goods few have broadened their conception or knowledge of a product to include service. (Gadrey, 2000) and (Agwu, 2014) pointed out that a product consists of both goods(tangible) and services(intangible). Product as any good service or idea that can be offered to a market to suit need, depending on the trans-situational (Helfenstein, 2005) goal of the individual this buttresses the point that anything produced and can satisfy the need of users is regarded to as a product. products can be either tangible or in tangible example of intangible products are travel, insurance, consulting and banking e.t.c for tangible products we have food, clothing, books and house e.t.c. Rathmell, (1966) opines that an economic product is a good or service he defined a good as tangible economic products that can be seen or touched and has the ability to satisfy needs. While Gadrey, (2000) defines a service as a product which perishes at it’s very instance of production, this describes that services are immaterial and intangible.

Product quality is the “totality” of features and characteristic of product or service that bear on its ability to satisfy implied or stated needs (Burril and Ledolter, 1999). Feigenbaum(1991) defines product quality as the “total” composition of product or service features of engineering, manufacturing, marketing, and through which in the product or service will meet the expectations of the consumers

The “total” composite product and service characteristics of marketing, engineering, manufacturing, and maintenance through which the product and service in use will meet the expectations of the customer - A. V. Feigenbaum. Quality is the totality of features and characteristics of products or service that bear on its ability to satisfy given needs. (American society for quality).All aspect of a product (good or service that bears on the ability to satisfy fitness for use, effectiveness and safety (Qualitydigest.com, 2015).

From the definition of Gadrey ,Rathmell and related other authors with similar definitions, that has described products as the totality of goods and service. That is why this research work centers on using product quality interchangeably with total quality.

**2.1.1 A BRIEF HISTORY OF QUALITY MANAGEMENT**

Quality management has become a key management function as a result of the increasing level of competition and dynamism in the business environment. According to Powell, (1995) total quality management’s origin can be traced to 1949, when the union of Japanese scientists and engineers came together and formed a committee of scholars, engineers and government officials dedicated to improving Japanese efficiency and enhancing their post-war quality of life and then American firms began to take seriously, total quality management around 1980.

Although it is not easy to say for sure the exact date the term total quality management came into existence, but it is clear that the term and philosophy came into existence around the mid 80’s. According to Bemowsky, (1992) the term total quality management was first established in 1985 by the naval air system command to describe its Japanese style management approach to quality improvement.

This concept was before known as total quality control, first used by the writer Feigenbaum in his first book on total quality control, a revision of the book originally published under the title ‘Quality control’ in 1951. He then defined it as an effective system for integrating the quality development, quality maintenance, and quality improvement efforts of the various groups in an organization so as to enable production and service at the most economical levels which allow for full customer satisfaction. The word ‘control’ was afterwards changed to ‘management’ which justifies the view of Crosby (1979) who stressed that control is not necessary when a zero defects level is achieved. The term “control” is at times understood as meaning control over the workforces activities, and this is clearly not the aim of TQM (Godfrey ,Dale, Marchington and Wilkinson, A 1997).

In the U.S.A the establishment of total quality management came as a result of the entrance of Japanese products into their markets to create competition which started in the 70’s, together with the impact of the study and writings of Deming, Juran, Crosby and Feigenbaum integrating their approaches with quality management birthed the concept, academics and companies studied the works of this authors and other scholars such as Ishikawa The use of this term was seen at the beginning of the 90’s when it was widespread reaching its climax in 1993. The need for top management leadership has its source in the U.S.A, arising from the Hawthorne studies (Roethlisberger and Dickson, 1939), the works of Maslow (1954), McGregor (1960) and Ouchi (1981). Japan developed its total quality management approach gradually from the end of the Second World War, at the end of the 70s and towards the beginning of the 80’s, Japanese emphasis and the achievement of some American writers established the concern about the value of quality management in the U.S.A and progressively to the other parts of the world.

**2.1.2 THE CONCEPT OF QUALITY**

In any product quality is measured by what a consumer gets out of a product, not what the supplier puts into the product (Drucker,1985). Quality in business can be defined as the dominance of something. It means fitness for use (Juran,1989). And this fitness is determined by the consumer of the product.

Deming (1950) defined quality as concentrating on the efficient production of the quality that the markets expects, and he linked quality to and management, “costs go down and production goes up as improvement of quality is accomplished by better management of design, engineering, testing and by improvement of processes. The consumers of a particular product for example OMO detergent may put focus on the quality of the product and the degree to which it can satisfy their needs and then compare it to other products produced by competitors e.g Ariel detergent or Sunlight, this influences the purchasing behavior of the customer. The manufacturers of a product might measure the conformance quality the reliability quality , the performance or degree to which the product was produced appropriately.

The American society for quality(ASQ) has defined quality as a subjective term for which each person has his or her own definition. In technical usage, quality can have two meanings:

1. The characteristics of a product or service that bear on its ability to satisfy stated or implied needs;
2. A product or service free of defect.

Quality management is aimed at not only on product quality, but also the means to achieve it. Quality management therefore uses quality assurance and control of processes as well as products to achieve more constant quality. (Beliwoje, 2010)

Below are five aspects of quality:

1. Producing: this means manufacturing or making something available.
2. Checking: it is a form of examination that it means to validate that something has been one in the accurate manner.
3. Quality control: this means controlling the quality procedure to make sure that the outcome is expected.
4. Quality management: directing, coordinating, planning and monitoring a business organization to make sure that its performance is optimized through assessment and enhancement.
5. Quality assurance: this implies that means that assurance is obtained that a product is satisfactory or will meet expectation.

**2.1.3 THE CONCEPT OF TOTAL/PRODUCT QUALITY MANAGEMENT**

In some studies quality management is also referred to as total quality management. Fred,(2012). Mustapa,Nizamettin, Selim and Mehves (2005), Opined that The concept of quality has been defined by different authors in different ways, although literature gurus has also contributed scholars such as Juran, Crosby, Deming, Garvin, Ishikawa and Figenbaum has defined this concept in diverse ways. Juran defined product/total quality management as a focus on a trilogy of quality planning, quality control, and quality improvement

(Mitra, 1987). Crosby,(1996) defined total quality management as conformance to specifications or requirements. He also identified that requirements are based on the needs of customers, he then brought up 14 steps to achieve a zero effect quality improvement strategy in order to achieve performance improvement. Quality is predictable degree of dependability and uniformity, suited to the market at a low cost (Deming 1986), he also identified the 14 principles of quality management this is to improve performance and productivity in any business enterprise.

Ishikwa (1985) also placed emphasis on the value product quality control has in the improvement on organizational or business performance, in this area he contributed by using a diagram called “cause and defect diagram” to analyse quality problems. The first user of total quality control was Figenbaum was the initial user of the total quality control in literature, he explained that quality is describes the concept of PQM as product/ service characteristics of marketing, engineering, manufacturing and maintenance through which the product /service in use will meet the expectation by the customer (Mitra, 1987).

Kanji and Wallace, (2000) stressed that total quality management is an organizational culture aimed at satisfaction of customers needs and wants through continuous improvement, this differs from one country to another and one industry to another, and rather has specific principles to be implemented , which will in turn yield to increased market share , grater return on investments and increased profit e.t.c , this explains the fact that managers are to make conscious effort in the organization of specific strategies and polices and ensuring that they are also followed in order to effectively achieve stated goals. Awareness of managers on the importance of total quality management, alongside business process reengineering and other continuous development technique was stirred by the benchmarking movement to seek out study, implement and improve on best practices (Zairi and Youssef, 1995).

The manufacturing firm were historically the originator of continuous improvement, it later then spread to the service sector.

- 95% of manufacturing companies and 70% of service companies have used one form or other of quality improvement programs and then 55% of American executives and 70% of Japanese executives use quality improvement information at least monthly (Olion and Rynes, 1991), (Rigby, 1998).
- In international survey conducted, on over 4000 managers in 15 countries it was indicated that the usage of TQM was approximately 60% in 1997 (Rigby, 1998). A Survey of TQM and continuous improvement programs indicates 12 common aspects: Committed leadership, adoption and communication of TQM, closer customer relationships, benchmarking, increased training, open organization, employee empowerment, zero defects mentality, flexible manufacturing, process improvement, and measurement (Powel, 1995).

In addition, to establish critical factors of total quality management, various studies have been carried out by various researchers and different instruments were developed by individual researchers and institutions some of which include the Malcolm Baldrige Award, EFQM (European Foundation For Quality Management), and the Deming Prize Criteria. Based on these studies, a wide range of management issues, techniques, approaches, and systematic experiential investigation have been brought up.

Hence, Saraph, Benson and Schroder, (1989) developed 78 items, which were classified

into eight(8) significant factors to assess the performance of total quality management in business organization.

These critical factors are:

1. Role of divisional top management and quality policy.
2. Role of the quality department.
3. Role of training.
4. Role of product and service design.
5. Role of supplier quality management.
6. Role of process management.
7. quality data and reporting.
8. Employee relations.

Quality is a key factor in achieving business success. Management has to outline the quality goals, quality policies and quality plans so that employees are continuously reminded the key factor is the customer and not the product (Besterfield, 1995). Because only product with standard quality will be patronized leading to an expansion in the customer base.

Hence Price and Gaskill (n.d) further developed three dimensions of quality which are

1. Product service dimension: this is the degree to which a customer is satisfied with a good or service rendered.
2. People dimension: this is the level in which a customer is pleased with the existing relationship between the people and the supplying firm.
3. Process dimension: this is a measure of the satisfaction of the supplier with the internal work process that are then used to produce goods and services to the final consumers

**2.1.4 Principles of Total Quality Management**

1. Management commitment:

Direct involvement by the top level managers in specific and important aspect or program of a business (institute of employee training and development, 2009). In quality management it includes (1) setting up and supervising a quality committee, (2) formulating and establishing quality policies and objectives, and making sure that the employees understand work in line with this set goals (3) providing resources and training, (4) overseeing implementation at all levels of the organization, and (5) evaluating and revising the policy in light of results achieved (united nations development programme, 2009).

1. Mutually beneficial supplier relation:

A business organization and its supplier are interdependent and a mutually cordial relationship enhances the ability of both to create value (David, 2005). Once this is done there would be open and free communication both parties and future plans will be mentioned each will establish development activities on how to achieve product quality.

1. Employee empowerment:

This involves the management practice of giving out information, incentives , and power with employees (Jacquiline, 2014) This will enable them take initiative and make decisions to work out problems and improve service and productivity (Ham, 2012).Empowerment is a management practice that is based on the idea of giving employees power, adequate resources, authority, opportunity, motivation, as well holding them answerable and responsible outcomes of their actions, will contribute to their competence and fulfillment and translate to improved overall business performance. (Amir Elnaga, 2014).

1. Detail based decision making:

The thought process of selecting a logical choice from the available options. When trying to make a quality decision, a person must evaluate the positives and negatives of each available option, and consider all the alternatives (Sloper, 2008)

For effective decision making, a person must be able to forecast the outcome of each option as well, and based on all these items, determine which option is the best for that particular situation.

1. Continuous improvement:

This is as an approach to work that thoroughly seeks to achieve incremental changes in processes in order to achieve improved quality and efficiency. (Rouse, 2014). This concept is the responsibility of every worker in all the department( finance, production, research and development and sales e.t.c) and not just a few, and this overall performance should be permanent

1. Customer focus:

The direction of an organization toward meeting the needs of its clients. Being customer focus is the most essential attribute a business can posses and it involves ensuring that all aspects of the company put its customers' satisfaction first (American Society For Quality, 2013). Customers are seen as a great resources to every business because their availability affects the growth of the business on the short and long run. So business should depend on their customer and understand their current and future needs and should meet the requirement to exceed those needs. Also, having a customer focus usually includes maintaining an efficient customer relations and service program (Intrnational Standard Oganisation, 2012).

**2.1.5 SMALL AND MEDIUM SCALE ENTERPRISES.**

Introduction

It has consistently been argued that for developing nations (Nigeria included ) to grow and catch up with other developing nations, there is the urgent need for a viable entrepreneurship model that would help tackle the alarming poverty rate, reduce unemployment, illiteracy, chronic diseases, maternal and infant mortality, reduce crimes, conflict, terrorism/insurgency, while at the same time promote growth of SMEs, wealth creation, enhance value reorientation, preserve the ecosystem from abuse and finally achieve sustainable economic development (National Economic Empowerment and Development Strategy, 2004, DFID, 2009). Akhuemonkhan, I. A et al (2013). A study done by the Federal Office of Statistics reveals that about 97% of all the businesses in Nigeria employs less than 100 employees, implying that 97% of all businesses in Nigeria are "small businesses". The SME sector provides, an average of 50% of Nigeria’s employment and 50% of its industrial output Indeed, there appears to be an agreement that the development of SMEs in Nigeria is a giant step towards building a vibrant and diversified economy

In addition, Business experts have identified that the corruption downsizing and neglect of the impact of SME to economic growth are factors that have lead to a stagnant economy. Taking note of the meaningful contributions that SME brings to the economy: creation of wealth stimulating real economic growth e.t.c as listed above, it becomes essential for the SMES sub-sector to be rejuvenated towards playing its roles as expected. The SME remains an accelerating moving vehicle for the transformation of Nigerian economy. Finally for the government to achieve its goals and other economic reforms for the benefits of the stakeholders and the masses at large there should be more focus on revitalizing the SME sub-sector (Nkechi, Ikechukwu EJ and Okechukwu, 2012).

**Small and Medium Scale Enterprises Defined**

There is no standard or universal definition of SMEs. According to Sharafat , Humayun, and Muhammad (2014), different authors define SMEs differently. Some of the authors defined them in terms of capital assets some defined it on the basis of skill and turnover. The definition of SMEs strictly depends on the level of development of the country. In most developed economies like the United States of America (USA),the U.K. and Canada the definition criterion adopted a mixture of annual turnover and employment levels.

Definitions changes from time to time depending on the level of advanced technology, changes in the price level and other factors.

Small business as defined by small business administration (SBA): SBA defines a small business concern as one that is independently owned and operated, is organized for profit, and is not dominant in its field. Depending on the industry, size standard eligibility is based on the average number of employees for the preceding twelve months or on sales volume averaged over a three-year period. Examples of SBA general size standards include the following:

- Manufacturing: Maximum number of employees may range from 500 to 1500, depending on the type of product manufactured;
- Wholesaling: Maximum number of employees may range from 100 to 500 depending on the particular product being provided;
- Services: Annual receipts may not exceed $2.5 to $21.5 million, depending on the particular service being provided;
- Retailing: Annual receipts may not exceed $5.0 to $21.0 million, depending on the particular product being provided;
- General and Heavy Construction: General construction annual receipts may not exceed $13.5 to $17 million, depending on the type of construction;
- Special Trade Construction: Annual receipts may not exceed $7 million; and
- Agriculture: Annual receipts may not exceed $0.5 to $9.0 million, depending on the agricultural product

The National Economic Reconstruction Fund (2005), defines small scale industries as those industries whose fixed asset and cost of new investment does not exceed N10 million. In the new industrial policy in Nigeria, Small scale enterprise are defined as those enterprise with total investment of between N100, 000 and N2 million excluding the cost of capital and including working capital.

However, the definition now in Nigeria depends on the classification of employee size and asset base (excluding land and building).

**Micro/Cottage enterprise:** This is a type of industry with total assets (excluding land and building) of not less than N5million working capital, but not more than 10 workers.

**Small Scale enterprise:** An industry with above N5million as total assets (excluding land and building) and not less than N50million including, with labor size of not less than 49 workers.

**Medium Scale enterprise:** This is an industry with assets (excluding land and building) not less than N50million as capital employed, but not more than N500million, with working size of 50 to 199 workers.

**Table 1.1**

| **S/N** | **SIZE CATEGORY** | **EMPLOYMENT** | **ASSETS(million)excl, land and building** |
| --- | --- | --- | --- |
| 1 | Micro enterprises | below 10 | Below 5 |
| 2 | Small enterprises | 10 to 49 | 5 to less than 50 |
| 3 | Medium enterprises | 50 to 199 | 50 to less than 500 |

**Categorization of SMEs based on the services rendered:**

Sectors controlled by SMEs:

1. Agro allied
2. Petrochemical and fertilizer
3. Chemicals
4. Plastic
5. Electrical and Communication (Electronic)
6. Paper and wood pulp
7. Food processing
8. Machinery and fabrication
9. Construction
10. Cosmetic
11. Textiles and non-woven
12. Metal
13. Tools
14. Polymer
15. Information Technology (business center)
16. Pharmaceutical.

**2.1.6 Nature of SMEs**

Before most large firms and multinational organizations get to where they are today, they all started small and for a firm to be classified as large scale, there will be some little changes in areas like technology, infrastructure, management style, and finance e.t.c. but why SME’s are regarded as small is because of some distinct characteristics it posses. According to A.O Adelaja (2011), below are the characteristics of small and medium scale enterprises.

i. They exist in the form of sole proprietorship and partnership, though some Could be registered as limited liability companies.

ii. Management structure is simple thus decision-making is easy. Ownership and management fuse together in one person or few individuals.

iii. Relationship between employer and employees is largely informal.

iv. They operate in many areas of economic activities e.g. manufacturing, transportation, communication, etc.

v. Majority is labour intensive, requiring more human per capital per unit of production.

vi. The technologies involved are always very simple.

vii. Limited access to financial capital, (suffer from inadequacy of collateral).

viii. They make greater use of local raw materials.

ix. They enjoy wide dispersal throughout the country providing a variety of goods and services.

**2.1.7 Neglect Of Small And Medium Enterprises In Nigeria.**

Attempts by developed and developing countries to eradicate poverty and unemployment

focuses on the growth of large enterprises, based on the traditional economy of scale. This theory is predicated on the postulation that, ‘big’ is ‘better’ while ‘small is bad’. Small enterprises were seen as old-fashioned and synonymous with technological and economic Backwardness. As luck would have it almost all the businesses that were at first small, eventually became large business enterprises. Most of the Multinational Corporations like Philips international of Netherland, Sony of Japan etc. started as family business ventures.

In Nigeria, there are indigenous enterprises such as Adebowale Electrical and JOAS Electrical Industry Limited that started as small outfits, importing finished products. The same thing is related to enterprises like Doyin Investment, Eleganza Nigeria Limited, Dangote Group of companies and Dantata Group of companies to mention just a few that commenced operations as prime movers of trades and imports before they diversified into manufacturing business.

**Life Cycle Of Small And Medium Scale Enterprise**

Business enterprise goes through stages as they evolve over time from the start of development, growth and then decline or closure, these enterprises change its uniqueness in each of these stages in a way that often requires skills, resources structures to manage them effectively.(Stokes, Wilson And Mador, 2010).

Life cycle models;

Greiner(1972) cited by stokes Wilson And Mador (2010), developed an early revolutionary and evolutionary model as regards the growth of business, he was of the motion that organizations evolve through various stages but move from one stage to another was precipitated by crises that led to more revolutionary change, if a firm could scale through a particular stage then it is ready for the next stage. For instance at the first stage of growth through creativity crisis of leadership arises, once this is resolved the next stage of growth through direction begins. By the last stage a more mutual management approach emphasizes teamwork and matrix style organizational structures, but Greiner was not able to predict what crisis might precipitate the move into another phase/stage.A more composite model by stokes and Wilson (2006) described the five stages

Stage 1: Concept Stage

At the start-up of a business it under goes through the process of conception and planning, this may involve a market test or a part time operation, it involves creative thinking, information gathering and networking.

Stage 2 : Development/Abort Stage

The business is launched and developed into a viable size, one determining factor here is the response of the customers to the product or service a sole proprietor manages the enterprise through his or her efforts this determines if the firm develops or abort. This stage is a vulnerable stage as the business statistics indicates that it is the youngest and smallest firms that have the high rate of closure.

Stage 3:Growth/Decline Stage

Once a business is started the management of internal process and people are often critical. The allotment of managerial tasks, recruitment of non-owner managers and the development of a functionally organized team are prerequisites to take a business through this process. there is supervision of the activities that may be carried out by family members and friends of small number of employees, before complete dependence is placed on it.

Stage 4 Maturity

Most surviving SME’s goes through a process of crises and then stability, it may at this stage use more sophisticated implements and machinery trying to keep up with advancing technologies , invariably it possess some of the characteristics of larger firms.

Stage 5 : Re-growth/Decline

Once an enterprise has gotten competitive advantage in the market place over its rival , profits or external investments may be available to explore. The curve by Stokes and Wilson proposed that investment may prompt a second period of growth. Without this period of second growth further growth maturity stage may remain stagnant or possibly decline as competition becomes more strengthened from rivals or new entrants.


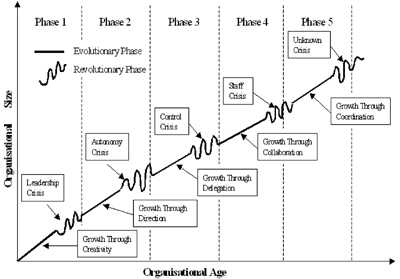


**Fig 1**The five phases of growth (adapted from Greiner, 1972)

**2.1.8 Relevance Of SME’s In The Economy.**

Small firms are backbone of national development. For a country to reach its full potential in terms of economic and social development, it cannot afford to ignore the importance of its small scale enterprise and the contributions that they make to the country’s economy, Osotimehin, Jegede, Akinlabi and Olajide (2014). The small and medium scale enterprise has a lot of impact to both developing and developed economies. They play critical roles and serve as the main engine of growth and a major factor in promoting private sector development and partnership.  SME’s are indeed the bases that provide the necessary drive for the transformation of most industrialized economies Agbo J.C Onu (2010).below are the vital relevance of SME’s in the economy.

**i. Technological/ industrial development**.

They have short-term gestation period and high potentials for quick yield on investment. They therefore provide promising alternatives for countries that desire the fast option for industrial development.

**ii. Employment Generation**

It has been observed that more jobs per unit of investment capital and per unit energy consumed are created worldwide by SMEs than large -scale enterprises

**iii. Technological Acquisition**

They provide opportunities for the development of local skills and technological acquisition. The "Aba made" syndrome is a clear manifestation of such technological acquisition and if encouraged will give rise to rapid economic development.

**iv. Innovation and Capacity building**

They provide a training avenue for the creation of local entrepreneurs in several areas of economic activities. They are regarded as the 'university' where a large class of Nigerian entrepreneurs usually receive training. It is therefore more important to lay a good foundation of an industrial class by enlightening youths and promoting start ups than it is to build a few large factories. and also improving the innovative abilities amongst the young and old in the economy, (Brock and Evans 1989).

**v. Promoting Growth**

Most are involved in primary and secondary economic endeavors that rely heavily on locally sourced materials, equipment and parts. As a result they achieve high local value added operators.

**vi. Increased standard of living**

Sound development of SMEs has positive impacts in improving the standard of living of the Citizens and generates foreign exchange for added development of the economy. The activities of small business firms have resulted in the mobilization of the resources of the environment and thereby improving on the standard of living of the populace. (Ayozie, 2011).

**vii. Industrial Dispersal or spread**

They could easily be located in rural areas because they can survive on rudimentary industrial

infrastructure. Consequently, they serve as major facilitators for industrial dispersal and rural

development and thus help in mitigating the rural-urban drift.

**viii. Serving of large -scale industries**

It helps to bring about local supply of goods and services and supply, some large firms who have to rely on the small scale operators for business success (Shokan, 1997). Raw material and goods are supplied to the large -scale industries while they engage in distribution of finished product from such industries to consumers. By working closely with SME’s can develop a new customer base which may not be accessible to traditional distribution networks (Akinyele, 2015).

**ix. Export promotion**

Most SMEs engaged in manufacturing serve as channels for import substitution and export promotion.

**x. Structural Transformation of rural areas.**

When they are cited in rural areas, they help to improve rural infrastructure and the living standard of the people. Social amenities such as: road, electricity, pipe-borne water, telecommunication facilities, etc, are attracted to the area as a result of the presence of SMEs in the community.

**xi. Flexibility**

According to stokes and Wilson (2010), a labour market characterized by a major participation of women, immigrants, young and old workers, and with considerable premium placed on flexible specialization, the relative adaptability and flexibility of the small enterprise comes into its own. They react swiftly to changes in the operating environment. With the presence of small and medium scale business in the economy there is provision for a good testing

ground for new products and growing production technique.

**xii. Low Take-off requirements**

Take-off capital requirements are low. Small-scale industrialization therefore widens the scope

for participation in industrial activities by individuals with limited capital. They are effective

instruments of mass participation in industrial development(Anyadike, Emeh and Ukah, 2012) (Anyadike Nkechi, 2012).

**2.1.9 QUALITY MANAGEMENT IN SMALL AND MEDIUM SCALE ENTERPRISES**

A lot has been written over the past couple of decades on various quality management models and techniques this can be applied in any business organization to improve operational, managerial and eventually financial performance through the emphasis on quality products/services and processes. (Jones, 2013). Over the years this models and techniques have taken form and labels and they continue to remain and remain over time in both small and large firms and in the dynamic business environment. Overtime the most prevalence of these models have been on quality and total quality models. Quality assurance can be described as the evaluation and certification of a second party (customer) or a third (an independent certification body) of a firms quality system, this is to demonstrate that a specific standard quality system have been met (Husband & Mandal, 1999).

The distinctiveness between quality assurance and total quality management is that TQM places emphasis on the business as a whole rather than just the quality system. Product quality involves a holistic approach to quality and adopts a quality a culture on quality and then spreads through the entire business or firm form the top level to the lower/non management employees, this concept of quality becomes the responsibility of every one and the customers are redefined to both internal and external entities. However TQM covers a broader definition of quality than quality assurance, and has therefore been seen as the next step for achieving growth. Although most small business never proceed to that level even after receiving certification. Research as shown that just a smaller percentage of SMEs.

**2.1.10 Product feature and Competitive advantage.**

From the generics strategies developed by porter (1980;1985) a firm can achieve competitive advantage through its unique product features which is a function of product differentiation (Drisu, Iyiola, and Ibiduni 2013). Product features involves the creation of unique product or services with value, which are able to satisfy the needs of customers, this futures can be the size the weight, additives accessories and materials (Bala, 2012). Furthermore, past studies have argued that product feature is a distinct way to achieve competitive advantage (Kotha and Orne, 1989; Baines and Langfield-Smith, 2003).

Based on these arguments this study expects a positive relationship between product features and competitive advantage.

Thus :

**H_1:_** There a significant relationship between product features and competitive advantage.

**2.1.11 Continuous Improvement and Business Continuity.**

Continuous improvement is known to be change that happens over a period of time, this can be in the reengineering process of improvement in goods and services to serve customers better (Sushil, 2013). Sushil, (2013), added that continuous improvement invariably is linked with continuity, because as businesses improves on their quality systems over time it leads to a more advantage over rivals thereby leading to a lasting organization. McPhee, (2009) Also stated that a key characteristics of continuity is development in services rendered and goods produced, as this should be imbibed in the organizational culture.

Based on the contribution of the above authors this study expects a positive link between continuous improvement and continuity in small business.

Thus:

**H_2:_** There is a link between continuous improvement and continuity in small business.

**2.1.12 Perceived Quality And Profitability.**

Perceived quality is the one of the first attribute that either retains customer or prevents repeat purchase by a customer, it is viewed as a customer’s perception of a good or service, it known to be served market evaluation of latest consumption experience, this has an overall outcome on customer satisfaction (Parasuraman, Zeithaml, and Berry1985), customer satisfaction is an outcome expressed by the consumer as a result of the degree of product performance, this customers satisfaction as an effect on the business profitability (Angelova and Zekiri, 2011), nowadays companies are looking for ways to improve in the perceived quality of their product which leads to the satisfaction of customers because customers satisfaction is not only based on the present experience but also on the past and future experience there by having an effect on profitability: (Kotler, 2000), (Anderson, Fornell, & Lehmann, 1994).

Based on the cited works in the above study this expects a positive relationship between perceived quality and profitability.

Thus:

**H_3:_** There is a significant relationship between perceived quality and profitability.

**2.1.13 Product Durability And Market Share**

When a consumer of a product derives, the expected value from a product before it begins to deteriorate, that product is said to be durable, thus the appearance of a product can be determined by consumer if it is durable or not (Blijlevens, Creusen, & Schoormans, 2009). The durability/ appearance of a product can create a chance of success in the market as result of increased customer base and market share. (Lewalski, 1988; Bloch, 1995; Hertenstein, Platt, and Veryzer, 2005; Yamamoto and Lambert, 1994; Chang and Wu, 2007). Also most often the larger market share of the firm results from loyal customer who have been able to make repeat purchase and influence the buying behavior of other customers because of the perceive durability of the product (Wu and Zhoa, n.d).

This above statement has showed an expected relationship between product durability and market share.

Thus:

**H_4:_** Product durability is a measure of market share.

**2.2 THEORITICAL FRAMEWORK**

**2.2.1 Deming’s Theory.**

William Edwards Deming was an American statistician, professor, author, lecturer and consultant. In Japan, from 1950 onwards, he taught top management how to improve design and service, product quality, testing, and sales through various methods, including the application of statistical methods. Deming has made significant contributions to management and in Japan's later reputation for innovative high-quality products and its economic power. He also established a sampling technique that is still used by the U.S department of census and bureau of labour statistics, He is regarded as having had more impact upon Japanese manufacturing and business than any other individual not of Japanese heritage. Dr. Edwards Deming taught that by adopting appropriate principles of management, organizations can increase quality and simultaneously reduce costs (by reducing waste, rework, staff attrition and litigation while increasing customer loyalty). The key is to practice continual improvement and think of manufacturing as a system, not as bits and pieces

1. create constancy of purpose toward improvement of product and service, with the aim of becoming competitive and stay in the businesses and then to provide jobs: this explains for small businesses achieve growth, there should be continuous improvement in various products/services that are offered to customers, this will lead to increased competitive position and also make more jobs available for individuals (example the youths) in the economy, since unemployment has even been a major concern in the economy, if jobs are available there will be reduced social vices and loitering of young individuals along the streets. Once this can be achieved there is more assurance in the continuity of the business. There should be long term plan for quality; there should be future prediction and challenges, with the striving goal of getting better.
2. Adopt the new philosophy. We are in a new economic age, western management must awaken to the challenge, must learn their responsibilities and take up leadership for change.

This principle suggests that, being in a very competitive and dynamic environment SME owners should always keep up with the trending technology, the dynamic change in customers taste and preferences and also should always try to be on top of the game, in embracing quality throughout the organization, notwithstanding in the prevailing economic system, in the area of changes in prices of materials, inflation rate, exchange rate, income earned by consumers e.t.c. The style of management in small business should be changed in such a way that there will be easy leadership, which will positively influence the employees and the business, a modern type of management system should be induced. The needs of the customers should be put at the top always, once this is done goals vision, will be implement with the aim of serving customers better.

1. Cease dependency on inspection to achieve quality, eliminate the need for inspection on a mass bases by building quality into the product in the first place.

If there is assurance on the quality of products, then there will be reduced emphasis on inspection of such product. Managers of small and medium enterprises should always seek quality first before quantity, quality should be constructed in products offered to customers, and this will lead to a greater return and then minimize cost on dependency on inspection. Quality should be inculcated from the start to finish in from the design of the product to the purchase of the product by the consumers. To then prove that the quality is right statistical control method not physical inspection alone.

1. End the practice of awarding business on the basis of price tag. Instead. Minimize total cost, move towards a single supplier for every one item, on a long term relationship of loyalty and trust.

Suppliers are major participants in the success of a business and quality leads to consistency, so therefore in the supply chain there should be a loyal supplier who supplies at a convenient price, these supplies should be looked at as partners in quality, there should be encouragement for them to improve in quality also. Quality statistics should be also used form time to time to access if the supplier’s quality also meets that of the business or that of the product yet to be produced.

1. Improve constantly and forever the system of production and service, to improve quality and productivity thus reduce cost.

There should be continuous improvement on processes and systems. Deming implemented the PLAN-DO-CHECK-ACT approach to process and analyze improvement.

Deming championed the work of Dr. Walter Shewhart which he called the shewhart cycle, and which has evolved into the PDSA cycle.

1. PLAN: The business owners should always drive and direct the quality process
2. DO: Not just planning and supervision, the SME participators should also participate in the quality process.
3. CHECK: There should be continuous review of the process intermittently
4. ACT: There should always be effective communication with the workers, recognition of their efforts and evaluation of the entire process.

**Figure 2**


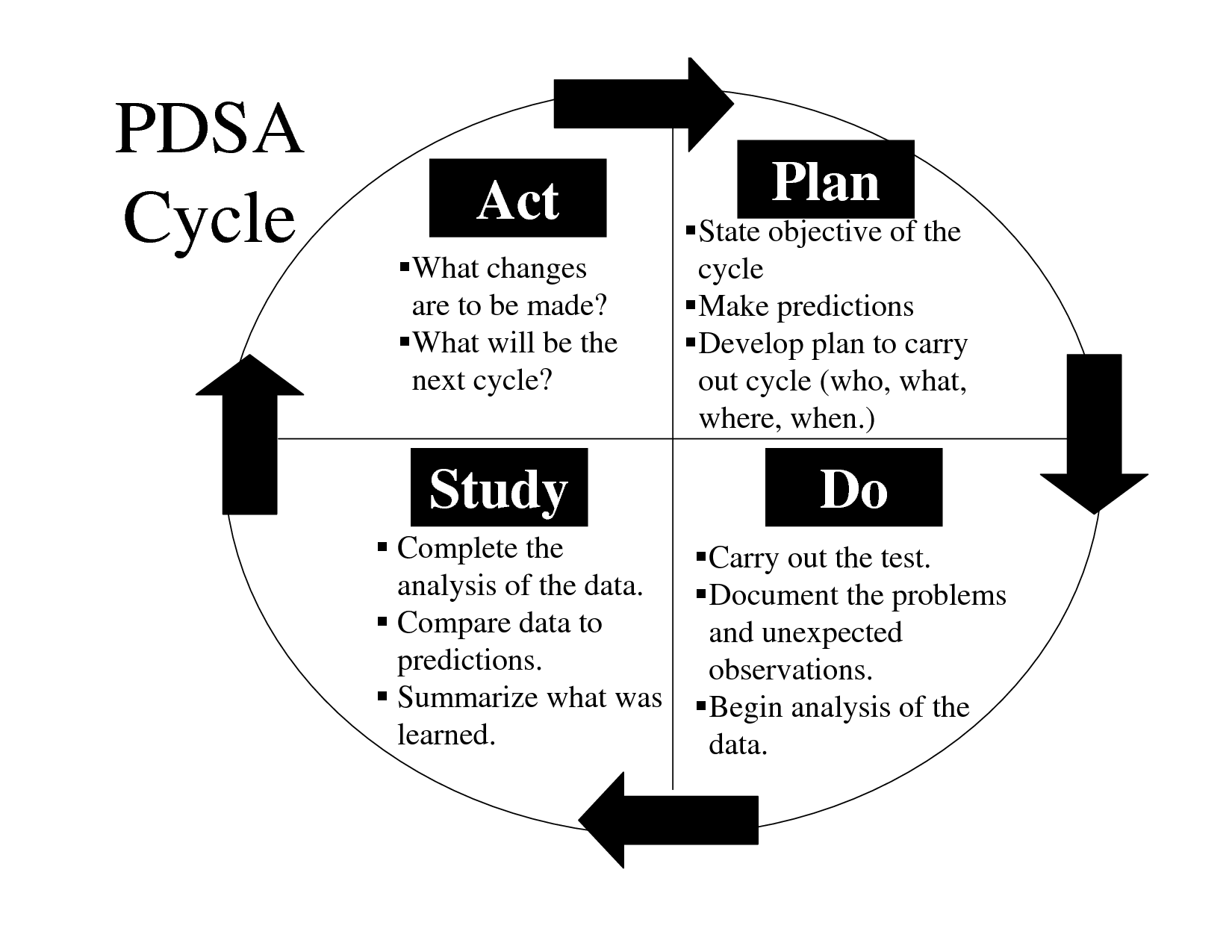


1. Institute training on the job.

SME’s owners or managers should adopt the method of continuous training, and not just training but modern method of training for all including the entire management this will lead to consistency and then reduce disparity. New skill will allow to keep up with the trending methods, materials and product design, machinery, techniques and services rendered, The foundation of common knowledge should be built, this entails allowing workers to understand their roles, there should be continuous encouragement from learning from each other, and a business culture and environment should be established for effective team work.

1. Institute leadership, the aim of supervision should be to organize machine and gadgets to perform better; supervision of management is in need of overhaul as well as supervision of management.

Leadership entails followership, if there should be effective followership the supervisors and managers should understand their workers and the processes they use, their roles is not just supervision but provision of adequate resource and support for effective output. their responsibility should be redirected form numbers to quality, there should be more emphasis on participative management and transformational leadership, the business managers should not just be interested in achieving target or goals but should seek ways to reach full potential, once this is done the quality imputed in the followers will spontaneously improve productivity.

1. Drive out fear so that everyone will work effectively for the company.

The business owners should ensure that the employees are not working under fear, they should ensure that there is a two way flow of communication where the workers are allowed to air their view and opinions this will help in the quality process, it becomes very futile if after training of employees the employees will not be allowed to contribute to the quality process. The managers themselves should be approachable and make the workers feel valued, and can work in teams to achieve the best results.

1. Break down barriers within departments; people in various departments such as research, design production must work as a team to achieve maximum result, to foresee problems of production and use that may be encountered with the product or service.

There should be no existence of barriers in such a way that the employees will understand the input of one department leads to the out put of another, for example the sales and marketing department cannot function without the production and finance department because the finance provides fund for the availability of raw materials which will be needed by the production department, the final product will the make marketing and sales possible. There should be more focus on collaboration and consensus instead of compromise.

1. Eliminate slogans, exhortations and targets for the work force that ask for zero defects and new levels of efficiency. Such exhortations only create adversarial relationships; the bulk of the causes of low quality and low productivity belong to the system, and thus lie beyond the power of the workforce.

Unclear slogans should be gotten rid of, “brilliance is service” this may be short and memorable but do the workers understand what is being communicated what is to be achieved how it is to be achieved, they should not be left to guess and deviate from intended goal, clear word like “you can achieve better if u try harder” should be used, this is well concise and not ambiguous.

1. Eliminate work standards (quotas) on the floor factory, substitute leadership. Eliminate management by objective, eliminate management by numbers, numerical goals substitute leadership.

Deming opined that production target encourages high output and low quality, small business owners should be more concerned about how the process is carried out and not just the numerical targets. There should be provision if adequate and quality resources so that production levels and are high and reachable. Although there are situations where bu MBO are appropriate to implement for example, in motivating sales person. As Deming already points out there are a number of situations where a focus on objective can lead to cut in quality.

1. Remove the barrier that rub the hourly worker of his right to enjoy the joy of workmanship, Remove barriers that rob people in management and in engineering of their right to joy of workmanship, this means abolishing the annual merit rating and abolishment of management by objective.

For quality system to improve, small business owners should ensure that there should be no existence of competition between employees for monetary or nonmonetary rewards, rather everyone should take pride in his or her work without comparison, the only form of competition that should exist should be between the company and other rivals in the market.

1. Institute a vigorous program of self improvement and education.

There should be continuous improvement in the skills of SME’s, their workers should encouraged to learn new skill and prepare for new changes, this will make the work force more adaptable to change and find means of better improvement in the business quality process.

1. Put everyone in the business to work to accomplish transformation this transformation is everyone’s job.

Transformation ought to be the job of everyone in the business, there should be overall improvement in the business quality, allowing each employee to take a step in achieving that, then those steps taken should be evaluated and see how it fits into each type of small business whether manufacturing, wholesaling retailing e.t.c and appropriate corrective measure should be taken.

**2.2.2 Tom Peters Management By Walking About (MBWA).**

Thomas J. “Tom Peters” An American writer on business management practices has had so many experience working as both a follower and then a leader, with is with his high level of qualifications he worked in the white house served in the US army and at some point also worked as a management consultant in Mckinsey and company. In his book in *“search for excellence* which he co-authored with Robert H. Waterman Jr. was where he encouraged *management by walking about* as the responsibility of managers to carry out.

This theory has been adopted by many managers in various organizations, by sampling events or employee discussions, which has facilitated improvement and morale, sense of organizational purpose, productivity and total quality management has been marked a success.

**Management By Walking About.**

Tom peters, identified that leadership is central to the process of quality improvement, disposing the word management for leadership. The key role of the leader as facilitator with the basis of Management By Walking About (MBWA), this allows the leader to keep in touch with , customers, suppliers, innovators and shareholders e.t.c.

Peters believes that as a manager performs this function three basic functions are happening.

1. Listening
2. Teaching
3. Facilitating

From the result that he carried out from successful business organisation, he concluded that any brilliant approach to managing had to incorporate and treat as interdependent, in what becomes the seven MCkinsey 7S framework. Whatever the type of change: restructuring, new processes, organizational merger, new systems, change of leadership, and so on, the model can be used to understand how the organizational elements are interrelated, and so ensure that the wider impact of changes made in one area is taken into consideration.

**Figure 3: Mckinsey Framework**


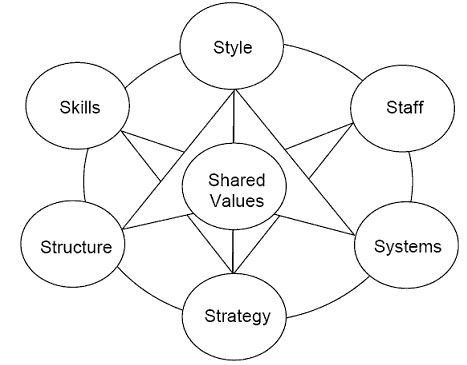


Source: http://www.valuebasedmanagement.net/methods_7s.html

This approach is designed to force explicit, the hardware and software part of any business they include. Structure, strategy, systems, shared values, skills, style and staff. (management.net, 2014) And relating it to the operations of small and medium enterprises.

- Structure: SME’s adopt this plan to maintain and also gain competitive edge over competitors by having a proper business structure: centralize, functional divisions, decentralized, matrix network e.t.c
- Strategy: this are plans for the proper allocation for scarce resources, over time to meet specific goals, this goals can be to achieve a better level of product quality.
- Systems:: this are specific procedures routines and processes, that character how it is important it is for work to be done this systems also birth subsystems. However small business no matter how small should have a reliable system: financial system, information system, promotion system e.t.c
- Shared values: this is a very important component because the shared values communicates what the organization stands for and what it believes in, SME’s manager should proper communicate this to employees in terms of quality, this will provide an understanding on how to work towards achieving desired goal.
- Skills: to achieve growth as a business employees and managers are expected to posses distinctive capabilities, to aid proper achievement of the best quality level.
- Style: this is the cultural style of small business and how managers believes in achieving product quality goals.
- Staff: the number and type of personnel available should be able to work together to achieve growth

This theory explains that for an organization to perform well, these seven elements need to be properly united and mutually revitalizing, this model can be used to determine and maintain what performance needs to be improved upon, and also to maintain alignment during other types of change. And since people (management) are a great assets to every both small or large, there should be proper coordination supervision and control of this 7S model this will enable better productivity, as managers makes sure they get feedback from both the internal and external environment on product quality level.

**2.2.3 Juran Quality Trilogy Theory**

Dr Joseph .M. Juran initiated the quality trilogy; quality planning, quality control and quality improvement. Proper quality management requires quality actions to be planned out, controlled and improved. This development achieves control at one level of quality performance, and then plans are made to improve the performance on a task by task basis, using appropriate tools and techniques such as the Pareto analysis. This activity eventually achieves a step forward to an improved level which is again controlled, to prevent any deterioration. Joseph Juran believed quality is associated with customer satisfaction and dissatisfaction with the product, and emphasized why it is essential for an on-going quality improvement through a succession of small improvement projects carried out throughout the firm.

**Figure 4: Juran Quality Trilogy.**

| **Quality control** | Holding the gains | **Quality planning** |
| --- | --- | --- |

Breakthrough Pareto Analysis

| **Quality improvement** |
| --- |

Project-by-project

Source: *www.dti.gov.uk/quality/gurus* the original quality gurus.

Juran’s ten steps to quality improvement are:

• Build awareness of the need and opportunity for improvement

• Set goals for improvement

• Organize to reach the goals

• Provide training

• Carry out projects to solve problems

• Report progress

• Give recognition

• Communicate results

• Keep score of improvements achieved

• Maintain momentum

He concentrated not just on the end customer, but on other external and internal customers. Each person along the chain, from product designer to final user, is a supplier and a customer. In addition, the person will be a process, carrying out some transformation or activity.

**2.3 EMPIRICAL FRAMEWORK**

The findings of an empirical survey of quality management in small and medium enterprises and an examination of how these are applied. This chapter summarizes the findings of different scholars and authors on quality management, total quality management or product quality management.

The possible contributions of studies demonstrated by Olusanya and Adegbola (2014), on the impact of Total Quality Management Practices on small and medium scale enterprises in Nigeria. The data used was based on primary data of questionnaire analysis that were distributed to small business owners in Lagos. from the distribution of 100 questionnaire to respondents, based on the findings is shows how challenges encountered in small business has led to a successful implementation of quality management practices in business organizations and also how the implementation of product quality has lead to a positive impact in small enterprises. Quality management here as opined by Olusanya and Agbola (2014), is not just aimed by ensuring “good quality” but rather to ensure that a business organization is consistent in whatever quality practice that is implemented, and this is buttressed to have four major components which are Quality assurance, quality planning , quality control and quality improvement, the concept of quality is not just on products but also a means to achieve it. Quality management uses quality assurance and control of all process and products to achieve more reliable quality (Lawal and Aikomo, 2008), this scholars in their study review the importance of quality management in organization which are: improvement in market share and overall productivity, improvement in product and service quality, bottom line improvement and increased number of satisfied customer, and all these poses as competitive advantage in the market place. Furthermore Because of the positive impact that quality management might bring firms (small and large) have embraced this in their operations, the level of the implementation of this concept seems to mirror a desire for the firm to improve performance and to prosper in achieving overall stated goals and objectives.

In another study carried out by Fening (2012), on the impact of quality management practices on the performance and growth of small and medium scale enterprises, this purpose of this research work was to investigate the implementation of quality management practices and how it has lead to growth in small businesses in a developing country. Based on the quantitative approach and the survey method of collecting data, 200 questionnaires was administered through face to face interview.

From the findings of this author it was established that if firms implement total quality management in their operations it will have a remarkable impact on the performance of SME. This study supports the fact that managers embrace the fact that quality management is a plus to their firms, it was also supported that the practice of quality management improves the overall performance of small and large firms globally.

Quality is said to help gain competitive advantage in firms that is a firm’s local product will have more recognition over other foreign products if quality management is taken seriously by a firm. Since managers will want to implement this concept, employees may know little or nothing about quality management, therefore communication which is very essential comes in play, in this regard careful implementation and attention ought to be given to the human resource (now human capital) and also leadership and more customer focus. The proper analysis of customer data must not be ignored as adequate information from customers will aid in proper direction of the firm in its production policy.

Finally it is therefore the implementation and practice of quality management or product quality management that firms will see the need to produce quality products. The practice of this concept is seen to be the responsibility of everyone in the organization and the government.

After all authors like by Olusanya and Adegbola (2014) researched on the impact of Total Quality Management Practices on small and medium scale enterprises in Nigeria, and by Fening (2012), on the impact quality management practices on the performance and growth of small and medium scale enterprises, and with other existing that researched on similar topic, this has shown there is a relationship between product quality management and growth of small and medium enterprise.

**2.4 GAP IN LITERATURE.**

One major goal for most businesses is to achieve growth and sustainability most managers sees this as a means of expansion and gaining competitive edge, this leads to so many benefits to the firms in areas of increased customer base and market share, increase in profit and general recognition of the firms product with this the need for quality management needs to be taken seriously. Based on the concept of quality management by several authors looking at several dimensions. The major gap in the practice of this study is that the implications of total quality in not just centered on what existing authors have mentioned but can be said to have an encompassing impact on the overall performance, the implementation of this concept definitely starts from one department to another and then yields to expansion of products this implies that once a product with quality is produced and has been appreciated by customers after proper evaluation and assessment of customers response to the product, firms can cease that opportunity to produce more differentiated products thereby serving a larger percentage of customers. Furthermore, the demand for the product would constantly ascend in the served markets and also get demand from countries that have not been directly entered into, leading to increased customer base and profit level of the organization, and hereby gaining sustainability and expansion.

Based on the theoretical review, most scholars place emphasis on the Deming theory of quality management, he said that by adoption of various principles of quality management can increase product quality and simultaneously reduce cost. In achieving quality the employees are to be involved in the overall implementation from top to bottom, with this it is seen that employees they are vital contributors to quality level, if this is properly understood the adoption of MBWA (management by walking around), this theory talks about how managers should take key interest in supervision and motivation of employees, and not just implementing quality management, once this is done everyone in the organization can work in unison to achieve product quality.

Finally one major gap is that several journals and literary work talks about the impact on quality management in organizational performance and the likes, but very few discussed on small and medium enterprises (SME’s), also most authors that talked about this concept are foreign authors, based on general search for literary works related to this area of study it was discovered that very few are form Nigeria.

**CHAPTER THREE**

**RESEARCH METHODOLOGY.**

**3.0 INTRODUCTION**

The Objective of this study was to show that product quality management, if well practiced has an impact on the growth of small businesses in Nigeria. This chapter describes the methodology that would be adopted to achieve the objective of this study.

It discusses the procedures for gathering data, sources of data, and the methods to be used in the data analysis.

**3.1 RESEARCH METHODOLOGY**

The methodology refers to the specification of procedures for collecting and analyzing data necessary in carrying out a research study (Smith and Albaum, 2010). It is a set of methods and principles used to perform a particular activity. Research methodology is the process used to collect information and data for the purpose of making decisions. The methodology used to carry out the research is the survey method. (Brikci and Green, 2007).The survey is a non-experimental, descriptive research method; surveys can be useful when a researcher wants to collect data on phenomena that cannot be directly observed. In a survey, researchers sample a population and this involves the use of questionnaires to gather information from the population. It includes the research design, the research instrument for collecting data, the level of validity and reliability and the method of presentation.

The non probability sampling technique was used to receive responses from respondents in Ikeja Lagos state.

**3.2 RESEARCH DESIGN**

Asika (1991), defined research design as the structuring of investigation aimed at identifying variables and their relationship to one another. Ojo (2003), also defines research design as a detailed plan of research that guides the researcher in the process in the process collecting, analyzing and reporting results.

The research for this work analyses the type of information collected the sources of data collection procedures used for the work. The study includes the methodology, population of the study, sample determination, the sample procedures, sources of data and data collection techniques adopted. The structure of this design is in such a way that it shows the relationship between the dependent and the independent variables, and the dimensions in which they are defined.

**3.3 POPULATION OF STUDY**

A population is the total group to be represented in a sample and which a researcher expects to be able to generalize the findings of the research. Barbie (1996), defined Research population as a census of all items or subjects that posses the characteristics or have knowledge of the phenomenon, being studied. The target population represented in this project is consists of the small business owners in Lagos state.

**3.4 SAMPLE SIZE DETERMINATION**

A sample is a subset of a population, which has the characteristics of the population and is studied in order to deduce a conclusion about the entire population. This study is carried out on a sample size of 368 employees drawn from the population; that is all workers in the organization. The sample size determination is crucial since there might be difficulties posed in studying the entire population. The sample size was therefore determined by using the Yard’s formula.

**Yard’s Formula**

The yard’s formula is a statistical formula which involves the application of normal approximation with 95% level of confidence and 5% error tolerance.

n = N

1+ a^2^N

Where N = population

n = sample size

a^2^ = level of significance

Therefore, in calculating the respondents that will complete the questionnaire, we have the followings;

N = 4,535 , n = ? , a = 0.05

n = 4,535

1 + (0.05)^2^ 4,535

n = 4,535

12.3625

n = 367.64

: Approximately 368.

Hair, Black, Babin and Anderson, (2010) argued that a sample size of between 100 and 200 is considered appropriate given a research involving a fairly large population, for this research work, 150 would be used for this project. Therefore the total number of respondents to be given questionnaires is 150.

**3.5 SAMPLE TECHNIQUE**

Sample technique can be explained as a particular and distinct way of getting samples from a target population. That is, techniques and procedures that are adopted to make provision for generalizing a population via a sample obtained from it. Sampling technques include probalility(simple random sampling, systematic, stratified and cluster sampling) and non probability tecnniques(judgemental, convience quote technique) (Abosede 2000). For this research, a probability sampling technique would be used in drawing the required sample size for the study. The procedure was chosen so as to guarantee randomness and equal representative by giving the respondent an equal chance of being selected. The statistical tool that would be used in analyzing the data will be the statistical packages for social sciences (SPSS). The non probability sampling technique of small business in Lagos State, constitute the techniques and procedures in the administration of the questionnaire.

**3.6 SAMPLE FRAME**

Sampling framing refers to the complete list of all units in the population under study and determines the structure of enquires (Olaseni, 2004). The sample frame is mainly the small business owners/ workers.

Below is the list of all SME’s in Nigeria, in various states

**Table 3.1**

TOTAL NUMBER OF SMALL AND MEDIUM ENTERPRISES BY STATE.

|  |  | EMPLOYEE SIZE BAND | | |  |  |
| --- | --- | --- | --- | --- | --- | --- |
| ITEMS | STATE | NUMBER(10-49) | PERCENTAGE | NUMBER(50-199) | PERCENTAGE | TOTAL |
| 1 | ABIA | 526 | 98.62 | 7 | 1.38 | 534 |
| 2 | ADAMAWA | 235 | 95.58 | 11 | 4.42 | 245 |
| 3 | AKWA-IBOM | 275 | 87.48 | 39 | 12.52 | 315 |
| 4 | ANAMBRA | 656 | 89.01 | 81 | 10.99 | 737 |
| 5 | BAUCHI | 497 | 91.02 | 49 | 8.98 | 545 |
| 6 | BAYELSA | 134 | 0 | 100.00 | 0.00 | 134 |
| 7 | BENUE | 357 | 95.63 | 16 | 4.37 | 374 |
| 8 | BORNO | 131 | 77.95 | 37 | 22.05 | 168 |
| 9 | CROSS RIVER | 318 | 87.02 | 47 | 12.98 | 365 |
| 10 | DELTA | 576 | 94.64 | 33 | 5.36 | 608 |
| 11 | EBONYI | 232 | 94.99 | 12 | 5.01 | 244 |
| 12 | EDO | 899 | 96.83 | 29 | 3.17 | 929 |
| 13 | EKITI | 280 | 98.41 | 5 | 1.59 | 285 |
| 14 | ENUGU | 402 | 93.03 | 30 | 6.97 | 432 |
| 15 | GOMBE | 225 | 88.02 | 31 | 11.98 | 255 |
| 16 | IMO | 534 | 92.97 | 40 | 7.03 | 574 |
| 17 | JIGAWA | 217 | 93.81 | 14 | 6.19 | 231 |
| 18 | KADUNA | 1,137 | 88.72 | 145 | 11.28 | 1,282 |
| 19 | KANO | 1,740 | 96.21 | 69 | 3.79 | 1,808 |
| 20 | KATSINA | 464 | 86.86 | 70 | 13.14 | 535 |
| 21 | KEBBI | 221 | 95.13 | 11 | 4.87 232 | 232 |
| 22 | KOGI | 328 | 96.67 | 11 | 3.33 | 340 |
| 23 | KWARA | 415 | 93.66 | 28 | 6.34 | 443 |
| 24 | LAGOS | 4,146 | 91.43 | 389 | 8.57 | 4,535 |
| 25 | NASSARAWA | 387 | 92.43 | 32 | 7.57 | 418 |
| 26 | NIGER | 433 | 90.48 | 46 | 9.52 | 478 |
| 27 | OGUN | 506 | 92.73 | 40 | 7.27 | 546V |
| 28 | ONDO | 596 | 97.13 | 18 | 2.87 | 614 |
| 29 | OSUN | 100 | 100.00 | 0 | 0.00 | 100 |
| 30 | OYO | 1,300 | 93.26 | 94 | 6.74 | 1,394 |
| 31 | PLATEAU | 613 | 92.56 | 49 | 7.44 | 663 |
| 32 | RIVERS | 662 | 91.65 | 60 | 8.35 | 723 |
| 33 | SOKOTO | 562 | 96.68 | 19 | 3.32 | 581 |
| 34 | TARABA | `242 | 97.80 | 5 | 2.20 | 247 |
| 35 | YOBE | 150 | 96.50 | 5 | 3.50 | 156 |
| 36 | ZAMFARA | 341 | 100.00 | 0 | 0.00 | 341 |
|  | FCT | 427 | 84.17 | 80 | 15.83 | 507 |
|  | TOTAL | 21,264 | 92.78 | 1,654 | 7.22 | 22,918 |

**Source: survey report on micro, small and medium scale enterprises in Nigeria (2010)**

**Table 3.1.1** Below is the relevant data extracted from the above list.

|  |  | EMPLOYEE SIZE BAND | | |  |  |
| --- | --- | --- | --- | --- | --- | --- |
| S/N | STATE | NUMBER | PERCENTAGE | NUMBER | PERCENTAGE | TOTAL |
| 24 | LAGOS | 4,146 | 91.43 | 389 | 8.57 | 4,535 |

**3.7 SOURCES OF DATA AND METHOD OF DATACOLLECTION.**

Information was obtained from both the primary and secondary sources during the research work.

**Primary sources**:

The primary sources of data employed in this study or research were questionnaires and personal observation. These primary sources include consumers and employees.

**Secondary sources**:

The secondary sources of data relied upon and used in this study were journals, textbook, past research work and marketing websites.

Data collection technique or procedure has two approaches which are the direct approach and the use of research assistants. Direct approach involves the researcher collecting the data himself when the respondents have finished filling out the questionnaires, while research assistants might be employed by the researcher to help him collect the data.

**3.8 RESEARCH INSTRUMENT AND DESIGN**

The research instrument to be used for the purpose of data collection is the Questionnaire.

According to Izedonmi (2005), a Questionnaire “is a research instrument used to elicit specific information (data) from respondents for a defined problem or study under investigation so as to gain better insight, understanding or appreciation of the issue being explored. It contains questions to which the respondents are expected to provide an answer.” Furthermore, Adedayo (2000) described a Questionnaire as ‘a set of questions, usually in printed for, which respondents are expected to respond to’.

The questionnaire will be administered personally by the researcher and collected personally to be able to get honest and not haphazard answers from the respondents. The questionnaire used in this study was designed in such a way that it will be easy for the respondents to respond to it. The research questionnaire is presented in to sections A and B and it was done to sample relevant information needed for testing the hypothesis. Section A contains the personal data of the respondent .i.e. Sex, Age, Marital Status, Educational Qualification and length of service While both section B contains five questions each, drafted to harvest the respondent view and opinion on the impact of product quality management on the growth of small businesses in nigeria. This questionnaire is constructed in simple and clear English for easy understanding.

**3.9 VALIDITY OF RESEARCH INSTRUMENT**

Validity is defined as the degree to which a measuring instrument measures what it is designed to measure (Ojo, 2003). Validity is the accuracy of the measurement. It is an evaluation of the accuracy of the depth relative to what actually exist (Ibidunni, 2010). The questionnaire was designed in such a way that information provided by the respondents would give valid answers to the research questions and hypothesis. The questionnaire has been certified valid by my supervisor and consultants who are experts in this field of study.

**3.10 RELIABILITY OF RESEARCH INSTRUMENT**

According to Osuagwu (2006) reliability can be defined as “the consistency between independent measurements of the same business research phenomenon.” It deals with the degree or extent to which a research instrument produces similar or consistent result overtime. Reliability is not only a necessary condition for validity; unreliable research measures lessen the correlation between research measures. Therefore, data on the research instrument were subjected to Cronbach alpha analysis to determine the reliability of the research instrument.

**Reliability Statistics**

**Table 3.2**

| Cronbach's Alpha | N of Items |
| --- | --- |
| .767 | 20 |

The result of the Cronbach’s alpha shows that the instrument used in generating responses from each of these categories of consumers measured what it intended to measure so the findings from it can be depended upon. It is reliable.

**3.11 METHOD OF DATA ANALYSIS**

The method of data presentation that will be used is the frequency table to allow for easy understanding and ability to grasp the result of findings in the research work. Research is meant to generate data for analysis and this usually results in outsized volume of statistical information, which is mostly in its raw stage. In order to use data for the objective of a research, they have to be cut down to manageable dimensions. Questionnaires are appropriately coded and edited for further data analysis via SPSS. Regression analysis would be used in the hypothesis testing. This shall be through the SPSS (statistical package for social scientist).

**CHAPTER 4**

**DATA PRESENTATION ANALYSIS AND INTERPRETATION**

**4.1 INTRODUCTION**

In the previous chapter, the research methods, process and sample size were discussed in details. The purpose of this chapter is to present the analyzed data and interpret the data obtained. The major aim of the research was to investigate the extent to which small and medium scale enterprises achieve growth through the application of product quality management practice. Small businesses in Ikeja was visited and 150 copies of questionnaires were randomly administered to small businesses, but a total of 137 questionnaires were returned fully and appropriately filled. Therefore the 137 copies received were used for the analysis. The questionnaires will be arranged, synthesized, edited and analyzed for reading comprehensive and making reliable conclusions.

**4.2 Presentation of Data**

The data gathered in this section covered the personal information of the respondents. This section of the questionnaire sought information on the respondents’ bio data and questions from the questionnaire. A simple analysis of the data collected is present.

**Table 4.1 Number of Respondents**

| **Questionnaires** | **Frequencies** | **Percentage %** |
| --- | --- | --- |
| Number returned | 137 | 91.33% |
| Number not returned | 13 | 8.66% |
| Total | 150 | 100% |

**Source: Field Survey (2015)**

In the table above, among 150 questionnaires that were administered, 137 (91.33%) of the respondents completely filled and returned the questionnaires administered to them, while 13 (8.66%) did not return their questionnaires.

**4.3 Data Analysis and Interpretations (Section A)**

**4.3.1 Socio-Demographic Characteristics of Respondents**

**Table 4.2 Gender of the Respondents**

|  | | Frequency | Percent | Valid Percent | Cumulative Percent |
| --- | --- | --- | --- | --- | --- |
| Valid | Male | 53 | 38.7 | 38.7 | 38.7 |
|  | Female | 84 | 61.3 | 61.3 | 100.0 |
|  | Total | 137 | 100.0 | 100.0 |  |

**Source: Field Survey (2015)**

Table 4.2 above shows the gender distribution of the respondents. Of the 137 respondents, 53 are male employees and 84 are female employees. This shows that more female respondents than male, this could also imply that there are more male employees than female.

**Table 4.3 AGE GROUP OF THE RESPONDENTS**

|  | | Frequency | Percent | Valid Percent | Cumulative Percent |
| --- | --- | --- | --- | --- | --- |
| Valid | 20 | 51 | 37.2 | 37.2 | 37.2 |
|  | 20-31 | 59 | 43.1 | 43.1 | 80.3 |
|  | 31-40 | 22 | 16.1 | 16.1 | 96.4 |
|  | 41-above | 5 | 3.6 | 3.6 | 100.0 |
|  | Total | 137 | 100.0 | 100.0 |  |

**Source: Field Survey (2015)**

Table 4.3 above shows that 51 (37.2%) are 20 and below, followed by 59 (43.1%) respondents between 20-31 years, 22 (16.1%) between 31-40 years and 5 are 41 years and above. These shows that the larger percentage of the respondents were between the ages of 20 to 30 years and this implies that majority of the sampled respondents are young entrepreneurs, and shows that youths are indulging more in entrepreneurship activities.

**TABLE 4.4 MARITAL STATUS OF RESPONDENTS**

|  | | Frequency | Percent | Valid Percent | Cumulative Percent |
| --- | --- | --- | --- | --- | --- |
| Valid | Single | 94 | 68.6 | 68.6 | 68.6 |
|  | married | 40 | 29.2 | 29.2 | 97.8 |
|  | Others | 3 | 2.2 | 2.2 | 100.0 |
|  | Total | 137 | 100.0 | 100.0 |  |

**Source: Field Survey (2015)**

Table 4.4 shows that 94 (68.6%) of the respondents are single, 40 (29.2%) are married, 3 (2.2%) are either divorced or widowed, this connotes that higher percentage of the respondents are single.

**TABLE 4.5 EDUCATIONAL QUALIFICATIONS OF RESPONDENTS**

|  | | Frequency | Percent | Valid Percent | Cumulative Percent |
| --- | --- | --- | --- | --- | --- |
| Valid | WASSCE | 30 | 21.9 | 21.9 | 21.9 |
|  | NCE/OND | 19 | 13.9 | 13.9 | 35.8 |
|  | HND/B.SC | 69 | 50.4 | 50.4 | 86.1 |
|  | PHD/MBA | 19 | 13.9 | 13.9 | 100.0 |
|  | Total | 137 | 100.0 | 100.0 |  |

**Source : Field Survey (2015)**

This table shows that 30 (21.9%) are WASSCE/OLEVEL holders, 19 (13.9%) are NCE/OND holders, 69 (50.4%) are HND/BSC holders, while 19 (13.9%) are PHD/MBA holder. This shows that most of the respondents are HND/BSC holders and implies that they are qualified learned personnel’s and therefore should have adequate knowledge of the topic being researched thereby valid giving valid responses.

**TABLE 4.6 YEARS OF EXPERIENCE OF RESPONDENTS**

|  | | Frequency | Percent | Valid Percent | Cumulative Percent |
| --- | --- | --- | --- | --- | --- |
| Valid | 2yrs | 43 | 31.4 | 31.4 | 31.4 |
|  | 2-5yrs | 56 | 40.9 | 40.9 | 72.3 |
|  | 6-10yrs | 26 | 19.0 | 19.0 | 91.2 |
|  | 11-above | 12 | 8.8 | 8.8 | 100.0 |
|  | Total | 137 | 100.0 | 100.0 |  |

**Source: Field Survey (2015)**

Table 4.6 above shows the duration at which the respondents have experience in businesses . The service years were grouped into different class intervals. 43 (31.4%) have 2 years of experience, 56 (40.9%) have 2-5 years of experience, 26 (19.0%) have 6-10 years of experience while 12 (8.8%) have 11 years of experience and above. This suggests that majority of the respondents are still young in the business and have more innovative ideas about the research topic.

**4.3.2 Summary of Socio-Demographic Characteristics of Respondents**

The bio data section of the questionnaire showed the respondents’ gender, age group, marital status, and educational qualification, years of experience in business. Most of the respondents are female and are single with educational qualification as HND/BSC holders, they fall within the age group of 20-31 years. A lot of others are between the ages of 20years and below and have 2-5 years of experience in the business.

**4.3.3 Test of Questionnaire**

**QUESTIONS ON THE EFFECT PRODUCT FEATURES HAS ON COMPETITIVE ADVANTAGE**

**4.7 THE ATTRIBUTES THAT OUR PRODUCTS POSSESS MAKES IT STAND OUT FROM THAT OF OUR RIVALS**

|  | | Frequency | Percent | Valid Percent | Cumulative Percent |
| --- | --- | --- | --- | --- | --- |
| VALID | STRONGLY DISAGREE | 1 | .7 | .7 | .7 |
|  | UNDECIDED | 5 | 3.6 | 3.6 | 4.4 |
|  | AGREE | 81 | 59.1 | 59.1 | 63.5 |
|  | STRONGLY AGREE | 50 | 36.5 | 36.5 | 100.0 |
|  | TOTAL | 137 | 100.0 | 100.0 |  |

**Source: Field Survey, 2015**

In Table 4.7 above, 50 (36.5%) respondents strongly agree, and 81 (59.1%) agree that the attributes of a product makes it stand out from that of rivals, while 5 (3.6%) respondents are undecided, none disagree and only 1 (.7%) respondent strongly disagrees with this. Majority of the respondents agree, thus, it can be concluded in small business that the attributes of a product makes it stand out from that of rivals.

**4.8 THE WIDE RANGE OF OUR PRODUCT, OFFERS DIFFERENT CHOICES FOR OUR CUSTOMERS TO CHOOSE FROM**

|  | | Frequency | Percent | Valid Percent | Cumulative Percent |
| --- | --- | --- | --- | --- | --- |
| Valid | strongly disagree | 1 | .7 | .7 | .7 |
|  | Disagree | 2 | 1.5 | 1.5 | 2.2 |
|  | Undecided | 6 | 4.4 | 4.4 | 6.6 |
|  | Agree | 72 | 52.6 | 52.6 | 59.1 |
|  | strongly agree | 56 | 40.9 | 40.9 | 100.0 |
|  | Total | 137 | 100.0 | 100.0 |  |

**Source: Field Survey (2015)**

In Table 4.8 above, 56 (40.9%) respondents strongly agree, and 72 (52.6%) the wide range of product, offers different choices for customers to choose from, while 6 (4.4%) respondents are undecided, 2 (1.5%) disagree and only 1 (.7%) respondent strongly disagrees with this. Majority of the respondents agree, thus, it can be concluded in small business the wide range of product, offers different choices for customers to choose from.

**4.9 THE SPECIAL FEATURES OF OUR PRODUCT HAVE INCREASED PATRONAGE**

|  | | Frequency | Percent | Valid Percent | Cumulative Percent |
| --- | --- | --- | --- | --- | --- |
| Valid | strongly disagree | 1 | .7 | .7 | .7 |
|  | Disagree | 1 | .7 | .7 | 1.5 |
|  | Undecided | 10 | 7.3 | 7.3 | 8.8 |
|  | Agree | 78 | 56.9 | 56.9 | 65.7 |
|  | strongly agree | 47 | 34.3 | 34.3 | 100.0 |
|  | Total | 137 | 100.0 | 100.0 |  |

**Source: Field Survey (2015)**

In Table 4.9 above, 47 (34.3%) respondents strongly agree, and 78 (56.9%) the special features of our product have increased patronage, while 10 (7.3%) respondents are undecided, only 1 (.7%) disagree and 1 (.7%) respondent strongly disagrees with this. Majority of the respondents agree, thus, it can be concluded that the special features of a product have increased patronage in SME.

**4.10 MAXIMIZING QUALITY, HAS LED TO A COMPETITIVE SUCCESS.**

|  | | Frequency | Percent | Valid Percent | Cumulative Percent |
| --- | --- | --- | --- | --- | --- |
| Valid | strongly disagree | 1 | .7 | .7 | .7 |
|  | Disagree | 2 | 1.5 | 1.5 | 2.2 |
|  | Undecided | 8 | 5.8 | 5.8 | 8.0 |
|  | Agree | 72 | 52.6 | 52.6 | 60.6 |
|  | strongly agree | 54 | 39.4 | 39.4 | 100.0 |
|  | Total | 137 | 100.0 | 100.0 |  |

**Source: Field Survey (2015)**

In Table 4.10 above, 54 (39.4%) respondents strongly agree, and 78 (56.9%) agree that Maximizing quality, has led to a competitive success, while 8 (5.8%) respondents are undecided, and 2 (1.5%) disagree and only 1 (.7%) respondent strongly disagrees with this. Majority of the respondents agree, thus, it can be concluded that Maximizing quality, has led to a competitive success, in SME.

**4.11 THE UNIQUE QUALITY OF OUR PRODUCT MEETS THE NEEDS AND PREFERENCES OF OUR CUSTOMERS**

|  | | Frequency | Percent | Valid Percent | Cumulative Percent |
| --- | --- | --- | --- | --- | --- |
| Valid | Disagree | 1 | .7 | .7 | .7 |
|  | Undecided | 13 | 9.5 | 9.5 | 10.2 |
|  | Agree | 66 | 48.2 | 48.2 | 58.4 |
|  | strongly agree | 57 | 41.6 | 41.6 | 100.0 |
|  | Total | 137 | 100.0 | 100.0 |  |

**Source: Field Survey (2015)**

In Table 4.11 above, 57 (41.6%) respondents strongly agree, and 66 (48.2%) agree that The unique quality of their product meets the needs and preferences of customers, while 13 (9.5%) respondents are undecided, and only 1 (.7%) disagree and none strongly disagrees with this. Majority of the respondents strongly agree, thus, it can be concluded that in SME unique quality of our product meets the needs and preferences of customers.

**QUESTIONS ON EFFECT OF CONTINUOUS IMPROVEMENT ON SMALL BUSINESS SUSTANABILITY.**

**4.12 CONTINUOUS IMPROVEMENT IN QUALITY IS SEEN AS A NECESSITY FOR THE BUSINESS TO ENJOY SUSTAINABILITY.**

|  | | Frequency | Percent | Valid Percent | Cumulative Percent |
| --- | --- | --- | --- | --- | --- |
| Valid | Undecided | 9 | 6.6 | 6.6 | 6.6 |
|  | Agree | 61 | 44.5 | 44.5 | 51.1 |
|  | strongly agree | 67 | 48.9 | 48.9 | 100.0 |
|  | Total | 137 | 100.0 | 100.0 |  |

**Source: Field Survey (2015)**

Table 4.12 above shows 67 (48.9%) respondents strongly agree, and 61 (44.5%) agree that Continuous improvement in quality is seen as a necessity for the business to enjoy sustainability, while 9 (6.6%) respondents are undecided, none disagrees and none strongly disagrees with this. Majority of the respondents strongly agree, thus, it can be concluded that Continuous improvement in quality is seen as a necessity for the business to enjoy sustainability.

**4.13 AN IMPROVEMENT IN THE PRODUCTION PROCESS ENSURES BETTER PRODUCTS**

|  | | Frequency | Percent | Valid Percent | Cumulative Percent |
| --- | --- | --- | --- | --- | --- |
| Valid | Disagree | 2 | 1.5 | 1.5 | 1.5 |
|  | Undecided | 7 | 5.1 | 5.1 | 6.6 |
|  | Agree | 60 | 43.8 | 43.8 | 50.4 |
|  | strongly agree | 68 | 49.6 | 49.6 | 100.0 |
|  | Total | 137 | 100.0 | 100.0 |  |

**Source: Field Survey (2015)**

Table 4.13 above shows 68 (49.6%) respondents strongly agree, and 60 (43.8%) agree that An improvement in the production process ensures better products, while 7 (5.1%) respondents are undecided, 2 (1.5%) disagrees and none strongly disagrees with this. Majority of the respondents strongly agree, thus, it can be concluded that An improvement in the production process ensures better products in SME.

**4.14 PROCESS IMPROVEMENT HAS HELPED THE COMPANY GROW IN TERMS OF CUSTOMER BASE**

|  | | Frequency | Percent | Valid Percent | Cumulative Percent |
| --- | --- | --- | --- | --- | --- |
| Valid | Disagree | 3 | 2.2 | 2.2 | 2.2 |
|  | Undecided | 14 | 10.2 | 10.2 | 12.4 |
|  | Agree | 72 | 52.6 | 52.6 | 65.0 |
|  | strongly agree | 48 | 35.0 | 35.0 | 100.0 |
|  | Total | 137 | 100.0 | 100.0 |  |

**Source: Field Survey (2015)**

In Table 4.14 above shows that, 48 (35.0%) respondents strongly agree, and 72 (52.6%) agree that Process improvement has helped the company grow in terms of customer base, while 14 (10.2%) respondents are undecided, 3 (2.2%) disagree and none strongly disagrees with this. Majority of the respondents agree, thus, it can be concluded that Process improvement has helped SME’s grow in terms of customer base.

**4.15 APPLYING THE SUGGESTIONS OF THE CUSTOMERS AND IMPROVING THE RIGHT ASPECT OF THE PRODUCT INCREASES CUSTOMER SATISFACTION**

|  | | Frequency | Percent | Valid Percent | Cumulative Percent |
| --- | --- | --- | --- | --- | --- |
| Valid | Disagree | 9 | 6.6 | 6.6 | 6.6 |
|  | Undecided | 6 | 4.4 | 4.4 | 10.9 |
|  | Agree | 58 | 42.3 | 42.3 | 53.3 |
|  | strongly agree | 64 | 46.7 | 46.7 | 100.0 |
|  | Total | 137 | 100.0 | 100.0 |  |

**Source: Field Survey (2015)**

In Table 4.15, shows that, 64 (46.7%) respondents strongly agree, and 58 (42.3%) agree that Applying the suggestions of the customers and improving the right aspect of the product increases customer satisfaction, while 6 (4.4%) respondents are undecided, 9 (6.6%) disagree and none strongly disagrees with this. Majority of the respondents strongly agree, thus, it can be concluded that Applying the suggestions of the customers and improving the right aspect of the product increases customer satisfaction.

**4.16 CONTINUOUS IMPROVEMENT IN THE BUSINESS'S PRODUCT LEADS TO PROFIT MAXIMIZATION**

|  | | Frequency | Percent | Valid Percent | Cumulative Percent |
| --- | --- | --- | --- | --- | --- |
| Valid | strongly disagree | 2 | 1.5 | 1.5 | 1.5 |
|  | Disagree | 5 | 3.6 | 3.6 | 5.1 |
|  | Undecided | 12 | 8.8 | 8.8 | 13.9 |
|  | Agree | 70 | 51.1 | 51.1 | 65.0 |
|  | strongly agree | 48 | 35.0 | 35.0 | 100.0 |
|  | Total | 137 | 100.0 | 100.0 |  |

**Source: Field Survey (2015)**

In Table 4.16, shows that, 48 (35.0%) respondents strongly agree, and 70 (51.1%) agree that Continuous improvement in the business's product leads to profit maximization, while 5 (3.6%) respondents are undecided, 5 (3.6%) disagree and 2 (1.5%) strongly disagrees with this. Majority of the respondents agree, thus, it can be concluded that in SME’s Continuous improvement in business's product leads to profit maximization.

**QUESTIONS ON EFFECT OF PERCEVIED QUALITY AND PROFITABILITY**

**4.17 THE QUALITY OF OUR PRODUCTS HAS INCREASED THE NUMBER OF COUNTRIES SERVED AND HAS INCREASED PROFIT MARGIN.**

|  | | Frequency | Percent | Valid Percent | Cumulative Percent |
| --- | --- | --- | --- | --- | --- |
| Valid | strongly disagree | 1 | .7 | .7 | .7 |
|  | Disagree | 14 | 10.2 | 10.2 | 10.9 |
|  | Undecided | 26 | 19.0 | 19.0 | 29.9 |
|  | Agree | 64 | 46.7 | 46.7 | 76.6 |
|  | strongly agree | 32 | 23.4 | 23.4 | 100.0 |
|  | Total | 137 | 100.0 | 100.0 |  |

**Source: Field Survey (2015)**

In Table 4.17, shows that, 32 (23.4%) respondents strongly agree, and 64 (46.7%) agree that The quality of products has increased the number of countries served and has increased profit margin, while 26 (19.0%) respondents are undecided, 14 (10.2%) disagree and 1 (.7%) strongly disagrees with this. Majority of the respondents agree, thus, it can be concluded that in small business The quality of products has increased the number of countries served and has increased profit margin.

**4.18 AN INCREASE IN THE QUALITY OF INPUT LEADS TO AN INCREASE IN THE QUALITY OF OUTPUT**

|  | | Frequency | Percent | Valid Percent | Cumulative Percent |
| --- | --- | --- | --- | --- | --- |
| Valid | strongly disagree | 2 | 1.5 | 1.5 | 1.5 |
|  | Disagree | 5 | 3.6 | 3.6 | 5.1 |
|  | Undecided | 12 | 8.8 | 8.8 | 13.9 |
|  | Agree | 74 | 54.0 | 54.0 | 67.9 |
|  | strongly agree | 44 | 32.1 | 32.1 | 100.0 |
|  | Total | 137 | 100.0 | 100.0 |  |

**Source: Field Survey (2015)**

In Table 4.18, shows, 44 (32.1%) respondents strongly agree, and 74 (54.0%) agree that An increase in the quality of input leads to an increase in the quality of output, while 12 (8.8%) respondents are undecided, 5 (3.6%) disagree and 2 (1.5%) strongly disagrees with this. Majority of the respondents agree, thus, it can be concluded that an increase in the quality of input leads to an increase in the quality of output.

**4.19 MAXIMIZING PROFIT THROUGH FOCUS ON QUANTITY IS MORE IMPORTANT THAN IMPROVING QUALITY**

|  | | Frequency | Percent | Valid Percent | Cumulative Percent |
| --- | --- | --- | --- | --- | --- |
| Valid | strongly disagree | 28 | 20.4 | 20.4 | 20.4 |
|  | Disagree | 29 | 21.2 | 21.2 | 41.6 |
|  | Undecided | 14 | 10.2 | 10.2 | 51.8 |
|  | Agree | 40 | 29.2 | 29.2 | 81.0 |
|  | strongly agree | 26 | 19.0 | 19.0 | 100.0 |
|  | Total | 137 | 100.0 | 100.0 |  |

**Source: Field Survey (2015)**

In Table 4.19, shows, 26 (19.0%) respondents strongly agree, and 40 (29.2%) agree that Maximizing profit through focus on quantity is more important than improving quality, while 14 (10.2%) respondents are undecided, 29 (21.2%) disagree and 28 (20.5%) strongly disagrees with this. Majority of the respondents agree, thus, it can be concluded Maximizing profit through focus on quantity is more important than improving quality, although the next majority are undecided and disagree on this statement.

**4.20 PARTICIPATION OF THE EMPLOYEES LEADS TO A SUCCESSFUL QUALITY MANAGEMENT PROCESS**

|  | | Frequency | Percent | Valid Percent | Cumulative Percent |
| --- | --- | --- | --- | --- | --- |
| Valid | Disagree | 7 | 5.1 | 5.1 | 5.1 |
|  | Undecided | 13 | 9.5 | 9.5 | 14.6 |
|  | Agree | 75 | 54.7 | 54.7 | 69.3 |
|  | strongly agree | 42 | 30.7 | 30.7 | 100.0 |
|  | Total | 137 | 100.0 | 100.0 |  |

**Source: Field Survey (2015)**

In Table 4.20, shows, 42 (30.7%) respondents strongly agree, and 75 (54.7%) agree that Participation of the employees leads to a successful quality management process, while 13 (9.5%) respondents are undecided, 7 (5.1%) disagree and none strongly disagrees with this. Majority of the respondents agree, thus, it can be concluded that in SME’s the Participation of the employees leads to a successful quality management process.

**40.21 AN INCREASE IN QUALITY LEVEL LEADS TO AN INCREASE IN COMPANY PROFIT.**

|  | | Frequency | Percent | Valid Percent | Cumulative Percent |
| --- | --- | --- | --- | --- | --- |
| Valid | strongly disagree | 1 | .7 | .7 | .7 |
|  | Disagree | 2 | 1.5 | 1.5 | 2.2 |
|  | Undecided | 8 | 5.8 | 5.8 | 8.0 |
|  | Agree | 68 | 49.6 | 49.6 | 57.7 |
|  | strongly agree | 58 | 42.3 | 42.3 | 100.0 |
|  | Total | 137 | 100.0 | 100.0 |  |

**Source: Field Survey (2015)**

In Table 4.21, shows, 58 (42.3%) respondents strongly agree, and 68 (49.6%) agree that An increase in quality level leads to an increase in company profit., while 8 (5.8%) respondents are undecided, 2 (1.5%) disagree and only 1 (.7%) strongly disagrees with this. Majority of the respondents agree, thus, it can be concluded that in An increase in quality level leads to an increase in company profit.

**QUESTION ON EFFECT OF PRODUCT DURABILITY ON MARKET SHARE**

**4.22 THE PRODUCT LIFE OF OUR PRODUCT HAS LED TO AN INCREASE IN CUSTOMER BASE.**

|  | | Frequency | Percent | Valid Percent | Cumulative Percent |
| --- | --- | --- | --- | --- | --- |
| Valid | strongly disagree | 1 | .7 | .7 | .7 |
|  | Disagree | 4 | 2.9 | 2.9 | 3.6 |
|  | Undecided | 17 | 12.4 | 12.4 | 16.1 |
|  | Agree | 75 | 54.7 | 54.7 | 70.8 |
|  | strongly agree | 40 | 29.2 | 29.2 | 100.0 |
|  | Total | 137 | 100.0 | 100.0 |  |

**Source: Field Survey (2015)**

In Table 4.22, shows, 40 (29.2%) respondents strongly agree, and 75 (54.7%) agree that The product life of our product has led to an increase in customer base, while 17 (12.4%) respondents are undecided, 4 (2.9%) disagree and only 1 (.7%) strongly disagrees with this. Majority of the respondents agree, thus, it can be concluded that The product life of our product has led to an increase in customer base.

**4.23 THE ENTIRE STAFF AND MANAGEMENT OF THE BUSINESS ORGANIZATION PARTICIPATE IN THE PRODUCTION OF QUALITY PRODUCTS**

|  | | Frequency | Percent | Valid Percent | Cumulative Percent |
| --- | --- | --- | --- | --- | --- |
| Valid | strongly disagree | 5 | 3.6 | 3.6 | 3.6 |
|  | Disagree | 8 | 5.8 | 5.8 | 9.5 |
|  | Undecided | 11 | 8.0 | 8.0 | 17.5 |
|  | Agree | 76 | 55.5 | 55.5 | 73.0 |
|  | strongly agree | 37 | 27.0 | 27.0 | 100.0 |
|  | Total | 137 | 100.0 | 100.0 |  |

**Source: Field Survey (2015)**

In Table 4.23, shows, 37 (27.0%) respondents strongly agree, and 76 (55.5%) agree that The entire staff and management of the business organization participate in the production of quality products, while 11 (8.0%) respondents are undecided, 8 (5.8%) disagree and 5 (3.6%) strongly disagrees with this. Majority of the respondents agree, thus, it can be concluded that that The entire staff and management of the business organization participate in the production of quality products.

**4.24 THE QUALITY OF OUR PRODUCTS HAS INCREASED THE REPUTATION OF YOUR BUSINESS**

|  | | Frequency | Percent | Valid Percent | Cumulative Percent |
| --- | --- | --- | --- | --- | --- |
| Valid | Disagree | 2 | 1.5 | 1.5 | 1.5 |
|  | Undecided | 11 | 8.0 | 8.0 | 9.5 |
|  | Agree | 71 | 51.8 | 51.8 | 61.3 |
|  | strongly agree | 53 | 38.7 | 38.7 | 100.0 |
|  | Total | 137 | 100.0 | 100.0 |  |

**Source: Field Survey (2015)**

In Table 4.24, shows, 53 (38.7%) respondents strongly agree, and 71 (51.8%) agree that the quality of the products has increased the reputation of your business, while 11 (8.0%) respondents are undecided, 2 (1.5%) disagree and none strongly disagrees with this. Majority of the respondents agree, thus, it can concluded that the quality of the products has increased the reputation of your business.

**4.25 THE LASTING NATURE OF OUR PRODUCT HAS MADE YOUR BUSINESS A MARKET LEADER**

|  | | Frequency | Percent | Valid Percent | Cumulative Percent |
| --- | --- | --- | --- | --- | --- |
| Valid | Disagree | 3 | 2.2 | 2.2 | 2.2 |
|  | Undecided | 25 | 18.2 | 18.2 | 20.4 |
|  | Agree | 72 | 52.6 | 52.6 | 73.0 |
|  | strongly agree | 37 | 27.0 | 27.0 | 100.0 |
|  | Total | 137 | 100.0 | 100.0 |  |

**Source: Field Survey (2015)**

In Table 4.25, shows, 37 (27.0%) respondents strongly agree, and 72 (52.6%) agree that the lasting nature of the product has made your business a market leader, while 25 (18.2%) respondents are undecided, 3 (2.2%) disagree and none strongly disagrees with this. Majority of the respondents agree, thus, it can concluded that the lasting nature of the product has made your business a market leader.

**4.26 OUR CUSTOMERS ARE SATISFIED WITH THE BENEFITS DERIVED FROM OUR PRODUCTS BEFORE IT BEGINS TO DETERIORATE.**

|  | | Frequency | Percent | Valid Percent | Cumulative Percent |
| --- | --- | --- | --- | --- | --- |
| Valid | strongly disagree | 1 | .7 | .7 | .7 |
|  | Disagree | 2 | 1.5 | 1.5 | 2.2 |
|  | Undecided | 15 | 10.9 | 10.9 | 13.1 |
|  | Agree | 63 | 46.0 | 46.0 | 59.1 |
|  | strongly agree | 56 | 40.9 | 40.9 | 100.0 |
|  | Total | 137 | 100.0 | 100.0 |  |

**Source: Field Survey (2015)**

In Table 4.26, shows, 56 (40%) respondents strongly agree, and 63 (46.0%) agree that the customers are satisfied with the benefits derived from our products before it begins to deteriorate, while 15 (10.9%) respondents are undecided, 2 (1.5%) disagree and only 1 (.7%) strongly disagrees with this. Majority of the respondents agree, thus, it can be concluded that the customers are satisfied with the benefits derived from our products before it begins to deteriorate**.**

**4.4 Test of Hypotheses and Discussion of Results**

The analysis of the data gathered has been carried out previously. Four sets of hypotheses are used for the purpose of this research, each of the hypothesis has both the null and alternate hypotheses. In testing and analysis of the hypotheses, the statistical test adopted is regression analysis.

Regression analysis establishes the effect several key variables identified in this study have on each other. Several hypotheses were formulated and tested through multiple regressions (ANOVA), one assumed to be the dependent variable and the other independent variables as stated in the model specified in the study.

The F ratio is calculated, this value is then compared with the value from F distribution table in our statistical table to determine the level of significance and at an appropriate degree of freedom.

R square is the coefficient of determination, the strength and direction of the relationship.

Beta determines the relative importance of the independent variable.

Standard errors show the strength of standard deviation. The higher the standard error, the less the significance.

T statistics is a measure that shows whether the relationship between the dependent and the independent variable is statistically significant. Sig T is the observed level of significance, for instance, a sig T of less or equal to 0.05 indicates a significant relationship.

**HYPOTHESIS 1**

**H_0:_** There is no significant relationship between product features and competitive advantage.

**H_1:_** There is a significant relationship between product features and competitive advantage.

**Table 4.27 Model Summary**

| Model | R | R Square | Adjusted R Square | Std. Error of the Estimate |
| --- | --- | --- | --- | --- |
| 1 | .671(a) | .450 | .446 | .43659 |

a Predictors: (Constant), product features

**Source Field Survey, 2015.**

Table 4.27 is the model summary. It shows how much of the variance in the dependent variable (competitive advantage) is explained by the model (product features). In this case the R square value is .450 expressed by a percentage, this means that our model (the effect product features has on competitive advantage) explains 45.0% of the variance on competitive advantage.

**Table 4.28 ANOVA(b)**

| Model |  | Sum of Squares | Df | Mean Square | F | Sig. |
| --- | --- | --- | --- | --- | --- | --- |
| 1 | Regression | 21.045 | 1 | 21.045 | 110.413 | .000(a) |
|  | Residual | 25.732 | 135 | .191 |  |  |
|  | Total | 46.777 | 136 |  |  |  |

a Predictors: (Constant), product features

b Dependent Variable: competitive advantage

**Source Field Survey, 2015.**

Table 4.28 shows the assessment of the statistical significance of the result. The ANOVA table tests the null hypothesis to determine if it is statistically significant. From the results, the model appears to have a good fit, indicated by positive F value of 110.413. Also, the table shows a statistically significant relationship between product feature and competitive advantage (p <0.01). The implication of the statistical result is that enhancing product features will result in a positive upward shift in firm’s competitive advantage. Hence the null hypothesis should not be accepted.

**Table 4.29 Coefficients(a)**

| Model |  | Unstandardized Coefficients | | Standardized Coefficients | T | Sig. |
| --- | --- | --- | --- | --- | --- | --- |
|  |  | B | Std. Error | Beta | B | Std. Error |
| 1 | (Constant) | .952 | .320 |  | 2.973 | .003 |
|  | Product features | .780 | .074 | .671 | 10.508 | .000 |

a Dependent Variable: competitive advantage

**Source Field Survey, 2015.**

Table 4.29 also shows which of the variables included in the model contributed to the prediction of the dependent variable. The study is interested in comparing the contribution of each independent variable; therefore beta values are used for the comparison. In this table, the beta co-efficient is .671, which relates to product features. It makes the strongest contribution to explaining the dependent variable. Hence we can say that there is a significant relationship between product features and competitive advantage.

**Interpretation of Results**

Findings from this result showed that there is a significant relationship between product features and competitive advantage.

We are accepting the alternative hypothesis (significant relationship between product features and competitive advantage) and not accepting the null hypothesis (there is no significant relationship between product features and competitive advantage).

**HYPOTHESIS 2**

**H_0:_** There is no link between continuous improvement and continuity in small business.

**H_1:_** There is a link between continuous improvement and continuity in small business.

**Table 4.30 Model Summary**

| Model | R | R Square | Adjusted R Square | Std. Error of the Estimate |
| --- | --- | --- | --- | --- |
| 1 | .508(a) | .258 | .253 | .54253 |

a Predictors: (Constant), continuous improvement

**Source Field Survey, 2015.**

Table 4.30 is the model summary. It shows how much of the variance in the dependent variable (small business sustainability) is explained by the model (continuous improvement). In this case the R square value is .258 expressed by a percentage this means that our model (effect of continuous improvement on small business sustainability) explains 25.8% of the variance on small business sustainability. The adjusted R square shows .253, while the standard error estimate indicates .54253 .

**Table 4.31 ANOVA(b)**

| Model |  | Sum of Squares | Df | Mean Square | F | Sig. |
| --- | --- | --- | --- | --- | --- | --- |
| 1 | Regression | 13.845 | 1 | 13.845 | 47.038 | .000(a) |
|  | Residual | 39.735 | 135 | .294 |  |  |
|  | Total | 53.580 | 136 |  |  |  |

a Predictors: (Constant), continuous improvement

b Dependent Variable: small business sustainability

**Source Field Survey, 2015.**

Table 4.31 shows the assessment of the statistical significance of the result. The ANOVA table tests the null hypothesis to determine if it is statistically significant. From the results, the model appears to have a good fit, indicated by positive F value of 47.038. Also, the table shows a statistically significant relationship between continuous improvement and small business sustainability (p <0.01). The implication of the statistical result is that continuous improvement of products will result in a positive upward shift in firm’s sustainability. Hence the null hypothesis should not be accepted.

**Table 4.32 Coefficients(a)**

| Model |  | Unstandardized Coefficients | | Standardized Coefficients | T | Sig. |
| --- | --- | --- | --- | --- | --- | --- |
|  |  | B | Std. Error | Beta | B | Std. Error |
| 1 | (Constant) | 1.713 | .388 |  | 4.418 | .000 |
|  | Continuous improvement | .620 | .090 | .508 | 6.858 | .000 |

a Dependent Variable: small business sustainability

**Source Field Survey, 2015**

Table 4.32 also shows which of the variables included in the model contributed to the prediction of the dependent variable. The study is interested in comparing the contribution of each independent variable; therefore beta values are used for the comparison. In this table, the beta co-efficient is .508, which relates to continuous improvement. It makes the strongest contribution to explaining the dependent variable. Hence we can say there is a link between continuous improvement and continuity in small business.

**Interpretation of Results**

Findings from this result showed that there is a link between continuous improvement and continuity in small business.

We are accepting the alternative hypothesis (there is a link between continuous improvement and continuity in small business).and not accepting the null hypothesis (there is no link between continuous improvement and continuity in small business).

**HYPOTHESIS 3**

**H_0:_** There is no significant relationship between perceived quality and profitability.

**H_1:_** There is a significant relationship between perceived quality and profitability.

**Table 4.33 Model Summary**

| Model | R | R Square | Adjusted R Square | Std. Error of the Estimate |
| --- | --- | --- | --- | --- |
| 1 | .183(a) | .033 | .026 | .80913 |

a Predictors: (Constant), perceived quality

**Source Field Survey, 2015.**

Table 4.33 is the model summary. It shows how much of the variance in the dependent variable (profitability) is explained by the model (perceived quality). In this case the R square value is .033 expressed by a percentage, this means that our model (effect of perceived quality on profitability) explains 3.3% of the variance on profitability. The adjusted R square shows .026, while the standard error estimate indicates .80913.

**Table 4.34 ANOVA(b)**

| Model |  | Sum of Squares | df | Mean Square | F | Sig. |
| --- | --- | --- | --- | --- | --- | --- |
| 1 | Regression | 3.056 | 1 | 3.056 | 4.667 | .033(a) |
|  | Residual | 88.382 | 135 | .655 |  |  |
|  | Total | 91.438 | 136 |  |  |  |

a Predictors: (Constant), perceived quality

b Dependent Variable: profitability

**Source Field Survey, 2015.**

Table 4.34 shows the assessment of the statistical significance of the result. The ANOVA table tests the null hypothesis to determine if it is statistically significant. From the results, the model appears to have a good fit, indicated by positive F value of 4.667. Also, the table shows a statistically significant relationship between perceived quality and profitability (p <0.01). The implication of the statistical result is that enhancing perceived quality will result in a positive upward shift in firm’s profitability. Hence the null hypothesis should not be accepted.

.

**Table 4.35 Coefficients(a)**

| Model |  | Unstandardized Coefficients | | Standardized Coefficients | T | Sig. |
| --- | --- | --- | --- | --- | --- | --- |
|  |  | B | Std. Error | Beta | B | Std. Error |
| 1 | (Constant) | 2.685 | .467 |  | 5.754 | .000 |
|  | Perceived quality | .248 | .115 | .183 | 2.160 | .033 |

a Dependent Variable: profitability

**Source Field Survey, 2015**

Table 4.35 also shows which of the variables included in the model contributed to the prediction of the dependent variable. The study is interested in comparing the contribution of each independent variable; therefore beta values are used for the comparison. In this table, the beta co-efficient is .183, which relates to perceived. It makes the strongest contribution to explaining the dependent variable. There is a significant relationship between perceived quality and profitability.

**Interpretation of Result**

Findings from this result showed that there is a significant relationship between perceived quality and profitability.

We are accepting the alternative hypothesis (There is a significant relationship between perceived quality and profitability).and not accepting the null hypothesis (There is no significant relationship between perceived quality and profitability).

**HYPOTHESIS 4**

**H_0:_** Product durability is not a measure of market share.

**H_1:_** Product durability is a measure of market share.

**Table 4.36 Model Summary**

| Model | R | R Square | Adjusted R Square | Std. Error of the Estimate |
| --- | --- | --- | --- | --- |
| 1 | .505(a) | .255 | .249 | .51519 |

a Predictors: (Constant), product durability

**Source Field Survey, 2015.**

Table 4.36 is the model summary. It shows how much of the variance in the dependent variable (market share) is explained by the model (product durability). In this case the R square value is .255 expressed by a percentage, this means that our model (effect of product durability on market share) explains 25.5% of the variance on market share. The adjusted R square shows .249, while the standard error estimate indicates .51519.

**Table 4.37 ANOVA(b)**

| Model |  | Sum of Squares | df | Mean Square | F | Sig. |
| --- | --- | --- | --- | --- | --- | --- |
| 1 | Regression | 12.249 | 1 | 12.249 | 46.150 | .000(a) |
|  | Residual | 35.831 | 135 | .265 |  |  |
|  | Total | 48.080 | 136 |  |  |  |

a Predictors: (Constant), product durability

b Dependent Variable: market share

**Source Field Survey, 2015.**

Table 4.37 shows the assessment of the statistical significance of the result. The ANOVA table tests the null hypothesis to determine if it is statistically significant From the results, the model appears to have a good fit, indicated by positive F value of 46.150. Also, the table shows a statistically significant relationship between product durability and market share (p <0.01). The implication of the statistical result is that enhancing product durability will result in a positive upward shift in firm’s market share. Hence the null hypothesis should not be accepted.

**Table 4.38 Coefficients(a)**

| Model |  | Unstandardized Coefficients | | Standardized Coefficients | T | Sig. |
| --- | --- | --- | --- | --- | --- | --- |
|  |  | B | Std. Error | Beta | B | Std. Error |
| 1 | (Constant) | 2.103 | .304 |  | 6.922 | .000 |
|  | Product durability | .497 | .073 | .505 | 6.793 | .000 |

a Dependent Variable: market share

**Source Field Survey, 2015**

Table 4.38 also shows which of the variables included in the model contributed to the prediction of the dependent variable. The study is interested in comparing the contribution of each independent variable; therefore beta values are used for the comparison. In this table, the beta co-efficient is .505, which relates to product durability. It makes the strongest contribution to explaining the dependent variable. There is a significant relationship between perceived quality and profitability.

**Interpretation of Results**

Findings from this result it shows that Product durability is a measure of market share. We are accepting the alternative hypothesis (Product durability is a measure of market share).and not accepting the null hypothesis (Product durability is not a measure of market share).

**4.5 Discussion of Results**

The statistical tests conducted in this chapter exposed that There is indeed a significant relationship between product features and competitive advantage as seen in the first hypothesis in which the linear regression was used for this hypothesis and showed the extent to which the variance in the competitive advantage can be explained by product future as 45.0% (.450) at 0.000 significant level, therefore the null hypothesis was not accepted.

For the second hypothesis test, the researcher tried to find out if there is a link between continuous improvement product and continuity in small business which was investigated through linear regression model. The model of the effect of continuous improvement on small business sustainability explained the extent to which it has an impact on small business continuity as 25.8 (.258) at 0.000 level of significance. Therefore the null hypothesis will not be.

The third hypothesis testing revealed there is a significant relationship between perceived quality and profitability which was investigated using linear regression model. The model of the effect of perceived quality on profitability explained the extent to which it enhances the total quality management process as explains 3.3% of the variance on profitability (.033) at 0.033 level of significance. Therefore the null hypothesis was not accepted.

Finally, the fourth hypothesis testing revealed that Product durability is a measure of market share which was checked through the linear regression model. The model of effect of product durability on market share explained the extent to which it enhances customer satisfaction with the product as explains 25.5% (.255) of the variance on market share at 0.000 level of significance. Therefore the null hypothesis was not accepted.

From the above findings, it can be concluded that product quality management in small and medium scale enterprise has an impact on their growth.

**Reliability Statistics**

**Table 4.39**

| Cronbach's Alpha | N of Items |
| --- | --- |
| .767 | 20 |

**Source Field Survey, 2015**

From the table above, Cronbach’s Alpha coefficient is .767 for the 20 items analyzed together. This shows that these items are highly reliable as they were more than the widely accepted score of 0.7 which indicated that the research instrument adopted is reliable.

**CHAPTER FIVE**

**SUMMARY AND CONCLUSION**

**5.0 Introduction**

This research was carried out with the primary objective of critically the impact of product quality management on the growth of small and medium scale enterprises in Nigeria. This chapter analyzes the summary of this research work as well as the result of the research having in veiw the findings, conclusions and recommendations made by the researcher based on the result of the data analysis from the field of study. This chapter also gives an overview of the whole research project as this starts with the summary of the work from Chapter 1 to Chapter 5. The findings that are in veiw are looked at from the theoretical and the empirical point of view as well as the conclusion of this research work. Recommendations that were proffered by the researcher in this project work were done in a way by which a benchmark was set for more researchers to build on this project work and to know what is expected from them as they carry out future studies. Also suggestions were made for further studies.

**5.1 Summary of Work**

According to various authors Olusanya and Adegbola (2014) supported by Fening (2012) Jones (2013), and various authors that researched on related topics argues that for businesses to remain on top of the game, in the competitive business environment, managers are compelled to adopt the best strategies to achieve growth and sustainability via product quality management. This research is aimed at analyzing the impact product quality management has on the growth of SME’s in Nigeria. This research is summarized according to the following chapters.

**In chapter one**, the topic was introduced in details, the statement of the problem, the objectives of the study, research questions and hypotheses were stated. Also, the significance of the research to small businesses, operationalization of research variables, methodology, scope of study, limitations, outline of chapters that will be discussed in the work and of course and the definition of major terms used during the course of presenting the chapter.

**Chapter two** of this study deals with the various reviews of literatures relevant to this research, which includes the conceptual framework; meaning of product quality as a totality of goods and services, history of product quality management, quality definition, concept of quality and product/total quality management, principles of total quality management, total quality management practices, concept definition and life cycle of SME’s, and the relationship between total quality management small and medium scale enterprises. It also consists of theories by different authors related to the study, and the empirical framework that went on to discuss the different contributions of authors related to the study and finally the gap in literature,

**In chapter three**, starting with the introduction of what the third chapter entails and then moved over to focus on the research methodology and it comprises of the research methods, research design, study population, sample size determination and sampling techniques, research sample frame, sources of data collection, research instruments, reliability of research and validity.

**Chapter four** has to do with the analysis of data gathered and the presentation of results as well as the source of various data and measurements used in the computations.

**Chapter five** is the final chapter in this study and it gives the summary and conclusions of this study. It also provides recommendations to other scholars and organizations.

**5.2 Findings**

This encompasses the analysis of all responses gathered on the field during the administration of questionnaires in the course of this study. The summary of findings is grouped into the theoretical and empirical findings.

**5.2.1 Theoretical findings**

This refers to the details and findings drawn from literatures used and other research findings.

From the Dewing’s (1950) theory where he initiated the 14 points of management, he taught that by appropriate application of management firms can achieve quality and reduce cost by eliminating waste, staff attrition and litigation.

Tom peters management by walking around he initiated this theory for manager to understand their roles as a facilitator, by replacing the word leadership with management, he opines that managers have three roles of (a) listening (b) teaching and (c) facilitating. He also initiated the 7S McKinsey framework, this model is used to enforce change and improvement in businesses.

Joseph Juran initiated the quality trilogy: quality planning, quality controlling and quality improvement, he emphasized that for proper quality practices proper plans should be initiated first by using appropriate tools and techniques such as the Pareto analysis, and then quality control methods which later moves to the final one of improvement, small businesses should always carry our continuous as it is the responsibility of everyone in the business, this will enable growth and survival in the changing business environment. with this he also built ten steps to quality improvement.

**5.2.2 Empirical Findings**

These findings were gotten from the data analyzed from the different sections in the aim to justify our hypotheses apart from that of the bio-data. These findings are itemized below as the following;

**Hypothesis One**

The statistical tests conducted in this chapter exposed that there is indeed a significant relationship between product features and competitive advantage this has also been proven by (Kotha and Orne, 1989; Baines and Langfield-Smith, 2003). Seen in the first hypothesis in which the linear regression was used for this hypothesis and showed the extent to which the variance in the competitive advantage can be explained by product future as 45.0% (.450) at 0.000 significant level, therefore the null hypothesis was not accepted.

**Hypothesis two**, the researcher tried to find out if there is a link between continuous improvement product and continuity in small business which was investigated through linear regression model. The model of the effect of continuous improvement on small business sustainability explained the extent to which it has an impact on small business continuity, this relationship has been established in the work of Sushil, (2013) as 25.8 (.258) at 0.000 level of significance. Therefore the null hypothesis will not be accepted.

**Hypothesis three** testing in this work and in the work of Angelova and Zekiri, (2011), revealed There is a significant relationship between perceived quality and profitability which was investigated using linear regression model. The model of the effect of perceived quality on profitability explained the extent to which it enhances the total quality management process as explains 3.3% of the variance on profitability (.033) at 0.033 level of significance. Therefore the null hypothesis was not accepted.

Finally, the **fourth hypothesis** testing revealed that Product durability is a measure of market share which was also revealed by Wu and Zhoa,( n.d) and checked through the linear regression model. The model of effect of product durability on market share explained the extent to which it enhances customer satisfaction with the product as explains 25.5% (.255) of the variance on market share at 0.000 level of significance. Therefore the null hypothesis was not accepted.

From the above findings, it can be concluded that product quality management in small and medium scale enterprise has an impact on their growth.

- 1. **Recommendations**

This study has adequately shown the impact of product quality management on the growth of small and medium scale enterprises and the following recommendations are proposed.

Small business should understand that the idea of product quality management is the responsibility of everyone in the organization and not just the worker in the manufacturing or in the human resource department alone. So everyone should embrace this ideology and work together towards achieving a maximum quality level. It is ultimate for small scale business to understand that small business success is the ability of the consumers to accept the product quality, and this success does not come in a day, therefore it is good for small business owners to understand that product quality, product features and product positioning should be continually improved to gain edge over competitors and to meet the needs and wants of consumers.

Since small business are seen as the engine of the economy in job creation and increasing the GDP, proper improvement in quality should be initiated in every business this therefore will lead to growth and survival in the economy. For best practices of quality management in businesses, managers should see training and development of managers and other employees in quality tools, principles, and techniques. This will increase performance and help in gaining competitive advantage over rivals. There should also what we call customer driven information, this implies that since customer are the target business seek to serve better, appropriate methods should be used to generate feedback from them as regards the performance of the purchased product, this will aid improvement and customer satisfaction. Business owners should know that, Meeting of customer needs and requirement , cost strategies , speed of delivery and flexibility is also part of the quality management improvement process, and not just focus on the product itself.

The principle of commitment should not be neglected by management, just because one techniques did not work, does not mean others would not work. Managers have to be self motivated in the practice of quality management, and also should embrace the idea of motivating employees via monetary and non monetary incentive s this will improve performance. Communication and corporation should be applied in small business, this will enable everyone to work together reduce conflict and lead to faster improvement.

**5.4 Conclusion**

The objective of this research is to critically examine the impact product quality management has on the growth of SME’s in Nigeria using small business owners in Lagos (ikeja) as case study. Therefore, based on the data gathered and analyzed and the results derived, this study has contributed to the existing body of knowledge concerning product/total quality management by measuring its impact on small business growth.

- 1. **Limitations of the Study**

One major limitation in the course of the study was time and inadequate information. This was due to the fact that not all employees who were given questionnaires returned them, as they were busy and have tight schedules. Also financial constraint is another factor, because of going tru and fro from Ogun state and Lagos to administer questionnaires.

Another major limitation in the course of carrying out this research is data constraint. Very few scholars have carried out research concerning this topic especially for the fact that it relates to small businesses which complicated the sourcing of relevant data.

- 1. **Contributions To Knowledge.**

Ultimately this research work has shown that product quality, leads to increased customer base, market share, increased profit level, good will, increased dominance over competitors and threat to larger industries, this research as proved a major way that small business can enjoy growth.

Furthermore, with the fact that few authors have researched on this topic, this work has contributed to the understanding of various concepts, definitions and theories used in this topic.

Finally, The study provides facts and figures that can be used by other researchers.

- 1. **Suggestions for Further Studies**

This research has presented product quality management as a major determinant of small and medium scale enterprises to achieve growth, using small business organizations as a case study.

However for further studies, it is suggested that there should be a comparative study between small and large firms that is the impact of product quality management on the growth of small and large firms. There can also be a study just on a particular type of firm for example on manufacturing industries.

**APPENDIX**

Department of Business,

College of Development Studies,

Covenant University,

KM, 10 Idiroko Road, Ota,

Ogun State.

Dear Respondent,

RE: QUESTIONNAIRE

IMPACT OF PRODUCT QUALITY MANAGEMENT ON THE GROWTH OF SMALL BUSINESSES IN NIGERIA.

I am an undergraduate of Business Administration in the Department of Business Studies, Covenant University, Ogun state. I am carrying out a research on the IMPACT OF PRODUCT QUALITY MANAGEMENT ON THE GROWTH OF SMALL BUSINESSES IN NIGERIA. This is in partial fulfillment of the requirements for an award of Bachelor of Science (B.sc) in Business Administration.

I kindly ask that you give all information needed for the completion of this research. Your response will be treated strictly as confidential, and for academic purpose. Please tick ( **√**) for Strongly agree(SA), Agree(A), Undecided(U),Disagree(D), and Strongly disagree(SD) as your choice from the alternative answers provided. Thank you.

Yours’ Faithfully,

Fadeke Anjorin

**SECTION A**

Please tick as appropriate and comment where necessary.

1. Gender: (a) Male ( ) (b) Female ( ).

2. Age: 20( ) 21-30( ) 31-40( ) 41 – above ( ).

3. Marital status: Single ( ) Married ( ) others, please specify ……………….

4. Educational qualification: WASSCE/ O LEVEL ( ), NCE/ OND ( ), HND/ B.SC ( ),

POST GRADUATE/ MBA ( ), OTHERS PLEASE SPECIFY…………

5. Length of service (a)Below 2yrs [ ](b)2-5yrs [ ](c)6-10yrs [ ](d)11yrs and above[ ]

**SECTION B**

**PART 1: THE EFFECT PRODUCT FEATURES HAS ON COMPETITIVE ADVANTAGE**

| No | ITEM | | SA | | | A | U | | | D | | | | SD | | | |
| --- | --- | --- | --- | --- | --- | --- | --- | --- | --- | --- | --- | --- | --- | --- | --- | --- | --- |
| 6 | The attributes that our products possess makes it stand out from that of our rivals | |  | | |  |  | | |  | | | |  | | | |
| 7 | The wide range of our product, offers different choices for our customers to choose from | |  | | |  |  | | |  | | | |  | | | |
| 8 | The special features of our product have increased patronage. | |  | | |  |  | | |  | | | |  | | | |
| 9 | Maximizing quality, has led to a competitive success | |  | | |  |  | | |  | | | |  | | | |
| 10 | The unique quality of our product meets the needs and preferences of our customers | |  | | |  |  | | |  | | | |  | | | |
| **PART 2: EFFECT OF CONTINUOUS IMPROVEMENT ON SMALL BUSINESS SUSTANABILITY** | | | | | | | | | | | | | | | | | |
| No. | ITEM | SA | | A | | | U | | | | D | | | SD | | | |
| 11 | Continuous improvement in quality is seen as a necessity for the business to enjoy sustainability. |  | |  | | |  | | | |  | | |  | | | |
| 12 | An improvement in the production process ensures better products. |  | |  | | |  | | | |  | | |  | | | |
| 13 | Process improvement has helped the company grow in terms of customer base. |  | |  | | |  | | | |  | | |  | | | |
| 14 | Applying the suggestions of the customers and improving the right aspect of the product increases customer satisfaction |  | |  | | |  | | | |  | | |  | | | |
| 15 | Continuous improvement in the business’s product leads to profit maximization |  | |  | | |  | | | |  | | |  | | | |
| **PART 3: EFFECT OF PERCEVIED QUALITY ON PROFITABILITY** | | | | | | | | | | | | | | | | | |
| No | ITEM | SA | | A | | | | U | | | | D | | | SD | |  |
| 16 | The quality of our products has increased the number of countries served and has increased profit margin. |  | |  | | | |  | | | |  | | |  | |  |
| 17 | An increase in the quality of input leads to an increase in the quality of output. |  | |  | | | |  | | | |  | | |  | |  |
| 18 | Maximizing profit through focus on quantity is more important than improving quality. |  | |  | | | |  | | | |  | | |  | |  |
| 19 | Participation of the employees leads to a successful quality management process. |  | |  | | | |  | | | |  | | |  | |  |
| 20 | An increase in quality level leads to an increase in company profit. |  | |  | | | |  | | | |  | | |  | |  |
| **PART 4: EFFECT OF PRODUCT DURABILITY ON MARKET SHARE** | | | | | | | | | | | | | | | | | |
| NO | ITEM | SA | | | A | | | | U | | | | D | | | SD | |
| 21 | The product life of our product has led to an increase in customer base. |  | | |  | | | |  | | | |  | | |  | |
| 22 | The entire staff and management of the business organization participate in the production of quality products |  | | |  | | | |  | | | |  | | |  | |
| 23 | The quality of our products has increased the reputation of your business. |  | | |  | | | |  | | | |  | | |  | |
| 24 | The lasting nature of our product has made your business a market leader |  | | |  | | | |  | | | |  | | |  | |
| 25 | Our customers are satisfied with the benefits derived from our products before it begins to deteriorates. |  | | |  | | | |  | | | |  | | |  | |

**REFERENCES**

# Bibliography

(n.d.).

(n.d.).

Adelaja, A. (2011). The Importance of Small and Medium Scale Industries in a Developing/Underdeveloped.

Akinyele, S. .. (2015, feburary 2). Promotin SSME'S for sustainable Development. ota, Ogun, Nigeria.

American Society For Quality. (2013, feburary 16). *ASQ.org.* Retrieved 2015, from ASQ Web Site: http://asq.org/learn-about-quality/total-quality-management/overview/overview.html

Amir Elnaga, A. I. (2014). Impact of employee empowerment on job satisfaction. *American journal of research communication, 2(1)*, 4-6.

Anderson, E. W., Fornell, C., & Lehmann, D. R. (1994, July). Customer satisfaction, market share and profitability: findings from sweden. *journal of marketing, 5*(3), 54-55.

Angelova, B., & Zekiri, J. (2011, september). Measuring Customer Satisfaction with Service Quality Using. *International Journal of Academic Research in Business and Social Sciences*, 233.

Anyadike Nkechi, E. I. (2012). Entrepreneurship development and employment. *universal journal of education and general studies, 4(1)*, 008-102.

Austrian Competition and Consumer Commission. (2012). SMALL BUSINESS AND THE COMPETITION AND CONSUMER ACT. In A. C. Commission, *SMALL BUSINESS AND THE COMPETITION AND CONSUMER ACT* (p. 15). Australia: Commonwealth of Australia.

Bala, H. (2012, december). The impact of small business management on product quality, features and product positioning in ibadan metropolitan. *International Journal of Psychology and Counselling, 4*, 136-142.

Bennett, D., & Vaidya, K. (2001). *Meeting Technology Needs of Enterprises for National Competitiveness.* Aston Business School. Vienna: UNITED NATIONS INDUSTRIAL DEVELOPMENT ORGANIZATION.

Blijlevens, J., Creusen, M. E., & Schoormans, J. P. (2009). How Consumers Perceive Product Appearance: the identification of three product appearance. *internation journal of design, 3*, 3.

Boulter, D. (2013). *STRATEGIC ANALYSIS FOR A SOFTWARE COMPANY IN THE CUSTOMER RELATIONSHIP MAMAGEMENT COMPANY.* Burnaby: DEPARTMENT OF BUSINESS ADMINISTRATION SIMON FRASER UNIVERSITY.

Brikci, N., & Green, J. (2007, FEBURARY). A GUIDE TO USING QUALITATIVE RESEARCH METHODS. *MEDECINS SANS FRONTIERS*, 2.

Burnett, J. (2010). *Introducing Marketing.* switzerland: a global text.

Collins, K. (2012, january). Magic Quadrant for Marketing Resource. *Gartar*, 26.

David, H. (2005). Principles For Driving Business Success. In H. David, *Factual Approach To Decision Making* (pp. 81-82). Act Publishers.

Dirisu, J. I., Iyiola, O., & Ibidunni, O. S. (2013, December). PRODUCT DIFFERENTIATION: A TOOL OF COMPETITIVE ADVANTAGE ND OPTIMAL BUSINESS PERFORMANCE. *Europeann Scientific Journal, 9*.

Fening, F. A. (2012, july). Impact of Quality Management Practices on the Performance and Growth of Small and Medium Sized Enterprises (Smes) in Ghana. *International Journal of Business and Social Science, 3*, 9-12.

Gadrey, J. (2000, september). THE CHARACTERIZATION OF GOODS AND SERVICES:. *46*(3), 370.

Ham, C. (2012). Leadership and engagement. *the king's fund*, 22.

Helfenstein, S. (2005, april). PRODUCT MEANING, AFFECTIVE USE EVALUATION,. *An Interdisciplinary Journal on Humans in ICT Environments, 1*(1), 77.

Husband & Mandal, P. (1999). Perceptions and Realities of Quality Methods in Australian Small- to Medium-sized Enterprises.Proceedings of the 12th Annual SEAANZ Conference, Victoria University of Technology. (pp. 143-145). Victoria University of Technology,.

institute of employee training and development. (2009). *strategic training.* australil: McGraw-Hill Australia.

Internatioal Monetary Fund. (2010, April). Rebalancing Growth. *Journal Economic And Financial Survey*, 15.

Intrnational Standard Oganisation. (2012). *Quality Mnagement Principles.* switzerlandd: ISO Central Secretariat.

Ivaničková, M. (2014). HUMAN RESOURCE MANAGEMENT AND HOFSTEDE'S MODEL OF SLOVAKIA. *journal of Comparative European Research*(2), 8.

Jacquiline, F. (2014, FEBURARY). EMPLOYEE EMPOWERMENT AND JOB SATISFACTION. *JOURNAL OF HUMAN RESOURSE DEVELOPMENT, 2*(2347-825X), 5.

Jones, M. X.-L. (2013). quality initiatives and business growth in austrian manafacturing SMEs: AN EXPLORATORY INVESTIGATION. *QUALITY MANAGEMENT AND SMALL BUSINSSES*, 11.

Lobo, M. X., & Jones, J. T. (n.d.). QUALITY INITIATIVES AND BUSINESS GROWTH IN AUSTRALIAN MANUFACTURING SMEs.

management.net, v. b. (2014). *7s framework.* management methods.

McPhee, I. (2009). *Business Continuity Management:Building resilience in public sector entities.* Australia: Australian National Audit Office.

Ogechukwu, A. D. (2011, december). The Role of Small Scale Industry in National Development in Nigeria. *Universal Journal of Management and Social Sciences, 1*.

Olusanya, S. O., & Adegbola, E. A. (2014, April). Impact of Total Quality Management Practice on Small and Medium Scale Enterprises in Nigeria.(A Case Study of Small Business Owners in Lagos). *Journal of Business and Management*(2278-487X), 1-7.

Oua, C. S., Liua, F. C., Hunga, Y. C., & C, D. Y. (2012). The Effects of Total Quality Management on Business Performance: Evidence from Taiwan Information-Related Industries. *journal of Decision Sciences and Management Information Systems*, 3-5.

Oua, C. S., Liua, F. C., Hunga, Y. C., & David, Y. C. (2012). The Effects of Total Quality Management on Business Performance: Evidence from Taiwan Information-Related Industries. *journal of Decision Sciences and Management Information Systems*, 3-5.

Rathmell, J. M. (1966, october). what is ment by service? *journal of marketing*, 32-33.

Rouse, M. (2014, Feburary 16). *Serch Manufacturing ERP.* Retrieved 2015, from Serch Manufacturing Web Site: http://searchmanufacturingerp.techtarget.com/definition/kaizen

Sloper, B. B. (2008). Understanding the dynamics of decision making and choice. *Social Policy Research Unit*(978-1-871713-24-4), 8.

Smith, S. M., & Albaum, G. S. (2010). *An Introduction to Marketing Research.* mexico: University Of New New Mexico.

Stepsis, J. A. (1998). *PARTICIPATORY MANAGEMENT:A NEW MORALITY.* Jossey-Bass/Pfeiffer.

Sushil. (2013, september). Does Continuous Change imply Continuity ? *Global Journal of Flexible Systems Managemen*, 1.

Teece, D. J. (2010). Business Models, Business strategy and innovation. *JOURNAL ON LONG RANGE PLANNING, 3*, 173.

united nations development programme. (2009). *HANDBOOK ON PLANNING,MONITORING AND EVALUATING EMPLOYEE DEVELOPMENT RESULTS.* new york: UNDP.

Wanjau, N. N., Kahiri, J., & Gakure, R. W. (n.d.). THE ROLE OF QUALITY IN GROWTH OF SMALL AND MEDIUM ENTERPRISES AND ECONOMIC DEVELOPMENT IN. *Department of Entreprenuership and Procurement, Jomo Kenyatta University of Agriculture and Technology, Nairobi,*, 459.

WU, L., & ZHAO, Y. (n.d.). LOYALTY AND DURABILITY: EVIDENCE FROM THE. *perking university journal*, 11-20.
